# Supplementary material for: Spin-related Cu-Co pair to increase electrochemical ammonia generation on high-entropy oxides
Source: Nat Commun. 2024 Jan 4;15:260. doi: 10.1038/s41467-023-44587-z (PMC10766993; doi:10.1038/s41467-023-44587-z)
Supplement: Supplementary file 1 — Supplementary Information [file 41467_2023_44587_MOESM1_ESM.pdf]

Supplementary Information

Title

Spin-related Cu-Co pair to increase electrochemical ammonia generation on high-entropy oxides

Authors

Shengnan Sun[1], Chencheng Dai[2,3], Peng Zhao[4], Shibo Xi[5], Yi Ren[1], Hui Ru Tan[1], Poh Chong Lim[1], Ming Lin[1], Caozheng Diao[6], Danwei Zhang[1], Chao Wu[5,7], Anke Yu[2], Jie Cheng Jackson Koh[2], Wei Ying Lieu[1,8], Debbie Hwee Leng Seng[1], Libo Sun[3,9], Yuke Li[4], Teck Leong Tan[4], Jia Zhang[4*], Zhichuan J. Xu[2*], Zhi Wei Seh[1*]

[1] Institute of Materials Research and Engineering (IMRE), Agency for Science, Technology and Research (A*STAR), 2 Fusionopolis Way, Innovis #08-03, Singapore 138634, Republic of Singapore. [2] School of Material Science and Engineering, Nanyang Technological University, 50 Nanyang Avenue, Singapore 639798, Republic of Singapore. [3] The Cambridge Centre for Advanced Research and Education in Singapore, 1 CREATE Way, Singapore 138602, Republic of Singapore. [4] Institute of High Performance Computing (IHPC), Agency for Science, Technology and Research (A*STAR), 1 Fusionopolis Way, #16-16 Connexis, Singapore 138632, Republic of Singapore. [5] Institute of Sustainability for Chemicals, Energy and Environment (ISCE^2), Agency for Science, Technology and Research (A*STAR), 1 Pesek Road, Jurong Island, Singapore 627833, Republic of Singapore. [6] Singapore Synchrotron Light Sources (SSLS), National University of Singapore, 5 Research Link, Singapore 117603, Republic of Singapore. [7] College of Materials Science and Engineering, Sichuan University, Chengdu, 610065, China. [8] Pillar of Engineering Product Development, Singapore University of Technology and Design, 8 Somapah Road, Singapore 487372, Republic of Singapore. [9] Department of Chemistry, City University of Hong Kong, Hong Kong SAR, P. R. China. These authors contributed equally: Shengnan Sun, Chencheng Dai and Peng Zhao. These authors jointly supervised this work: Jia Zhang, Zhichuan J. Xu and Zhi Wei Seh.

*E-mail: zhangj@ihpc.a-star.edu.sg; xuzc@ntu.edu.sg; sehzw@imre.a-star.edu.sg

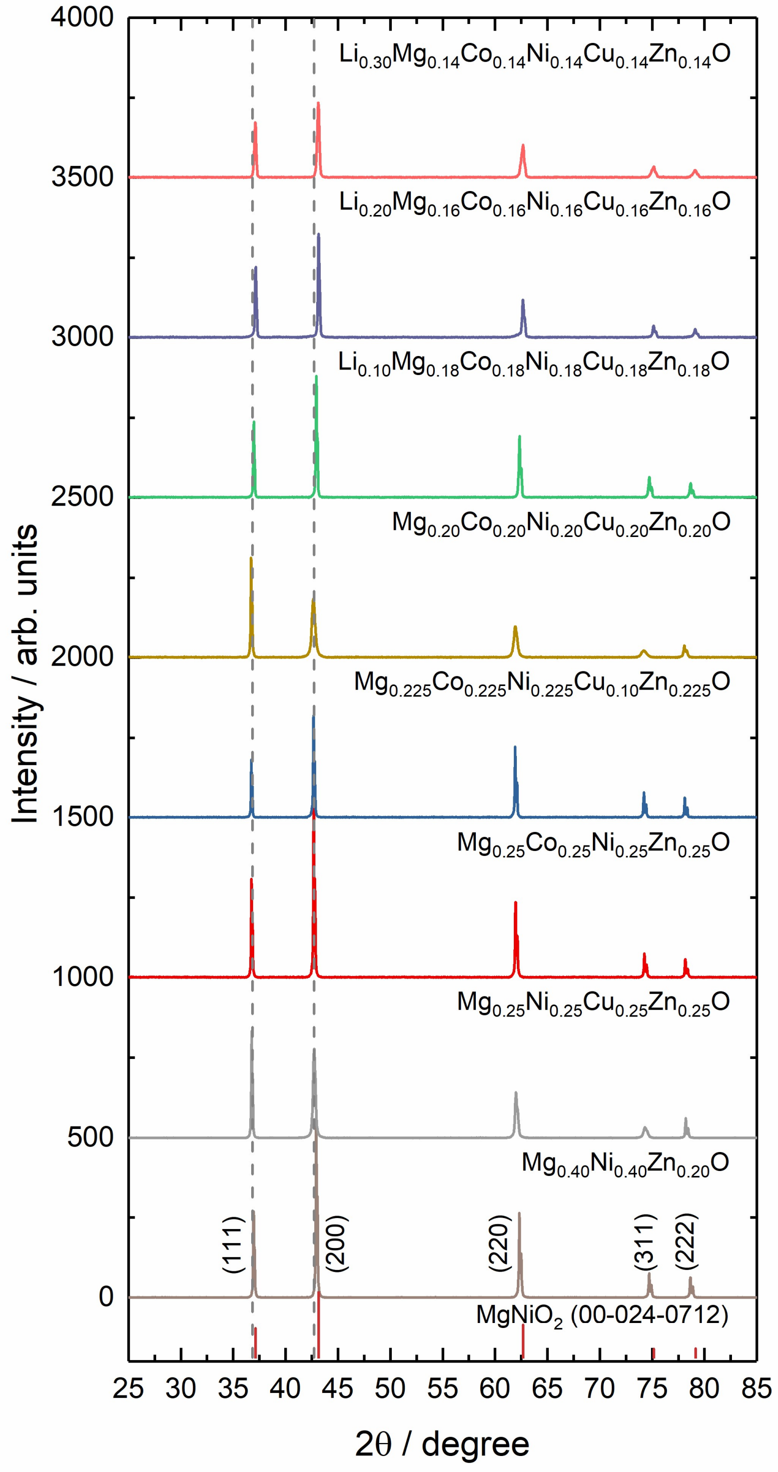

Supplementary Fig. 1 XRD patterns of $Mg_{0.40}Ni_{0.40}Zn_{0.20}O$ (MNZO), $Mg_{0.25}Ni_{0.25}Cu_{0.25}Zn_{0.25}O$ (MNCZO), $Mg_{0.25}Co_{0.25}Ni_{0.25}Zn_{0.25}O$ (RS-0), $Mg_{0.225}Co_{0.225}Ni_{0.225}Cu_{0.10}Zn_{0.225}O$ (RS-10), $Mg_{0.20}Co_{0.20}Ni_{0.20}Cu_{0.20}Zn_{0.20}O$ (RS-20), $Li_{0.10}Mg_{0.18}Co_{0.18}Ni_{0.18}Cu_{0.18}Zn_{0.18}O$ (Li-RS-18), $Li_{0.20}Mg_{0.16}Co_{0.16}Ni_{0.16}Cu_{0.16}Zn_{0.16}O$ (Li-RS-16) and $Li_{0.30}Mg_{0.14}Co_{0.14}Ni_{0.14}Cu_{0.14}Zn_{0.14}O$ (Li-RS-14).

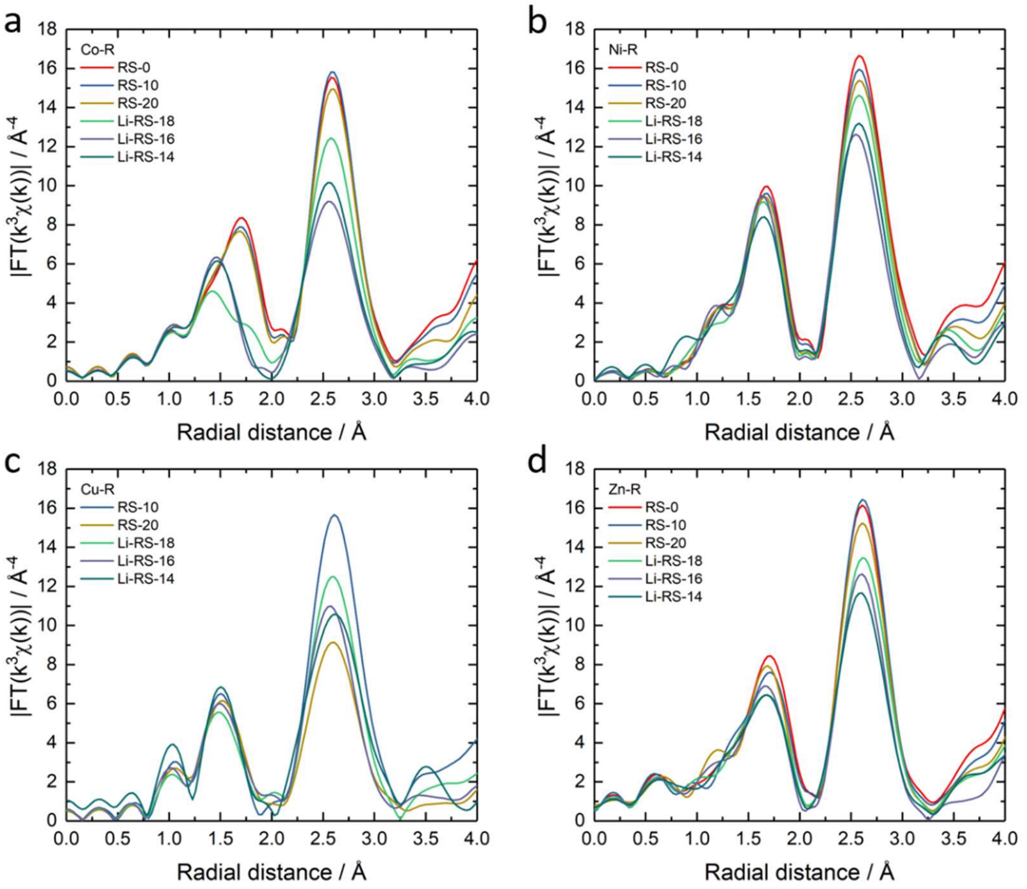

Supplementary Fig. 2 EXAFS of (**a**) Co, (**b**) Ni, (**c**) Cu, and (**d**) Zn of RS-0, RS-10, RS-20, Li-RS-18, Li-RS-16 and Li-RS-14.

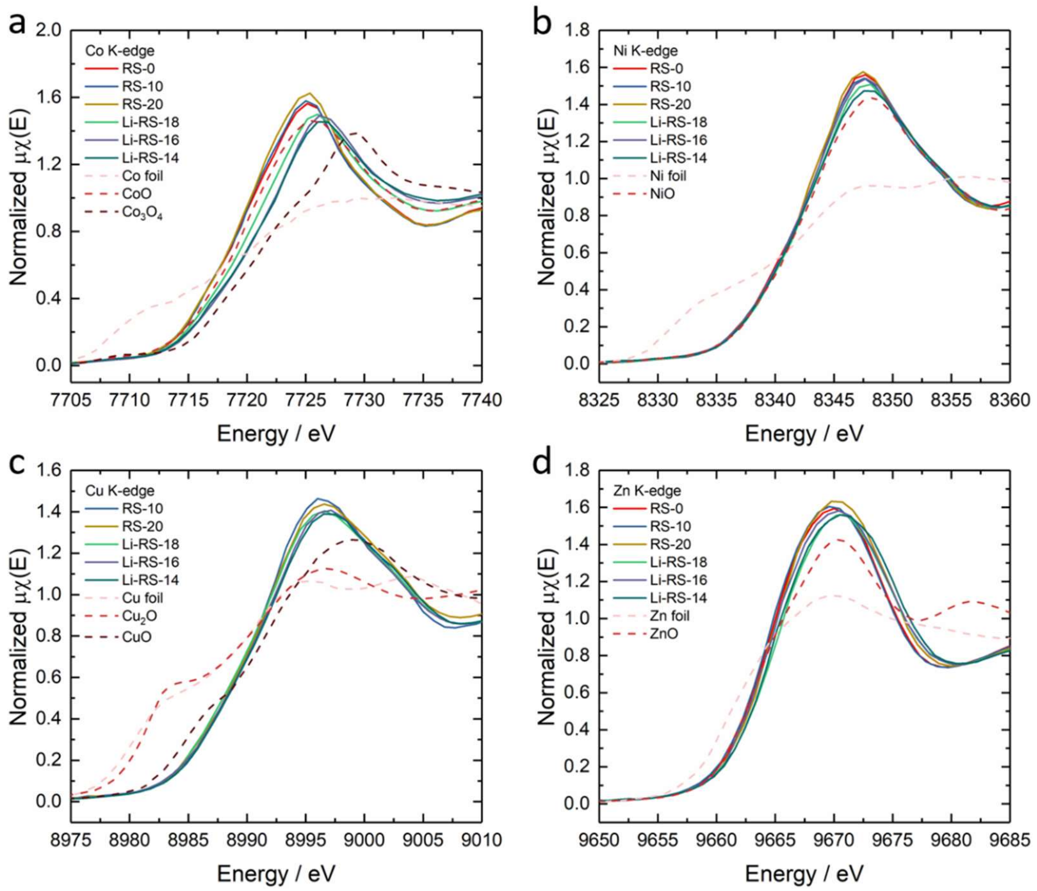

Supplementary Fig. 3 XANES of (**a**) Co, (**b**) Ni, (**c**) Cu and (**d**) Zn K-edges of RS-0, RS-10, RS-20, Li-RS-18, Li-RS-16, Li-RS-14 and the corresponding reference samples.

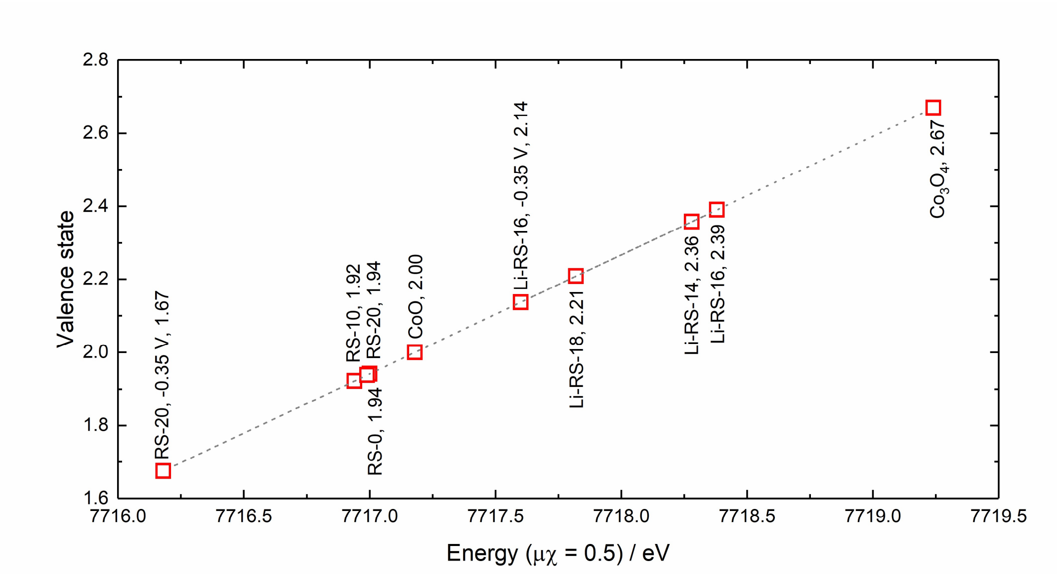

Supplementary Fig. 4 Estimated Co valence states of each sample from the linear fitting of valence states and energies (μχ = 0.5) of CoO and Co_3O_4 XANES.

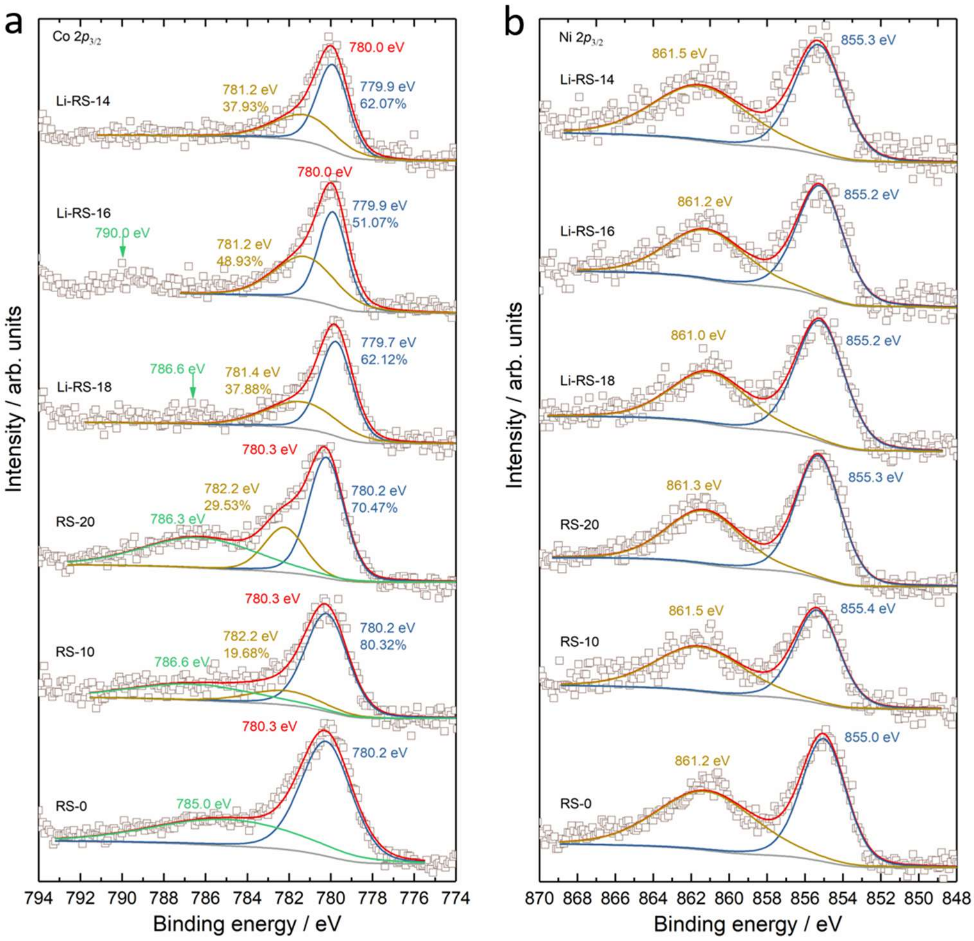

Supplementary Fig. 5 XPS results of (**a**) Co and (**b**) Ni in RS-0, RS-10, RS-20, Li-RS-18, Li-RS-16, and Li-RS-14.

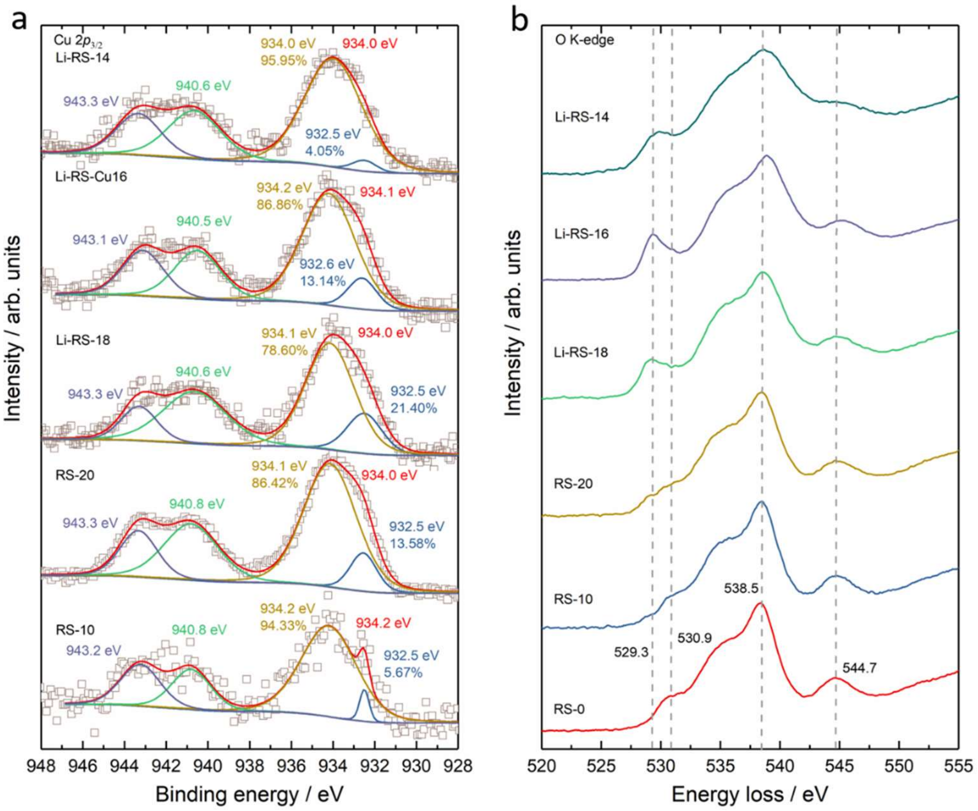

Supplementary Fig. 6 XPS results of (**a**) Cu and EELS measurement of (**b**) oxygen in RS-0, RS-10, RS-20, Li-RS-18, Li-RS-16, and Li-RS-14.

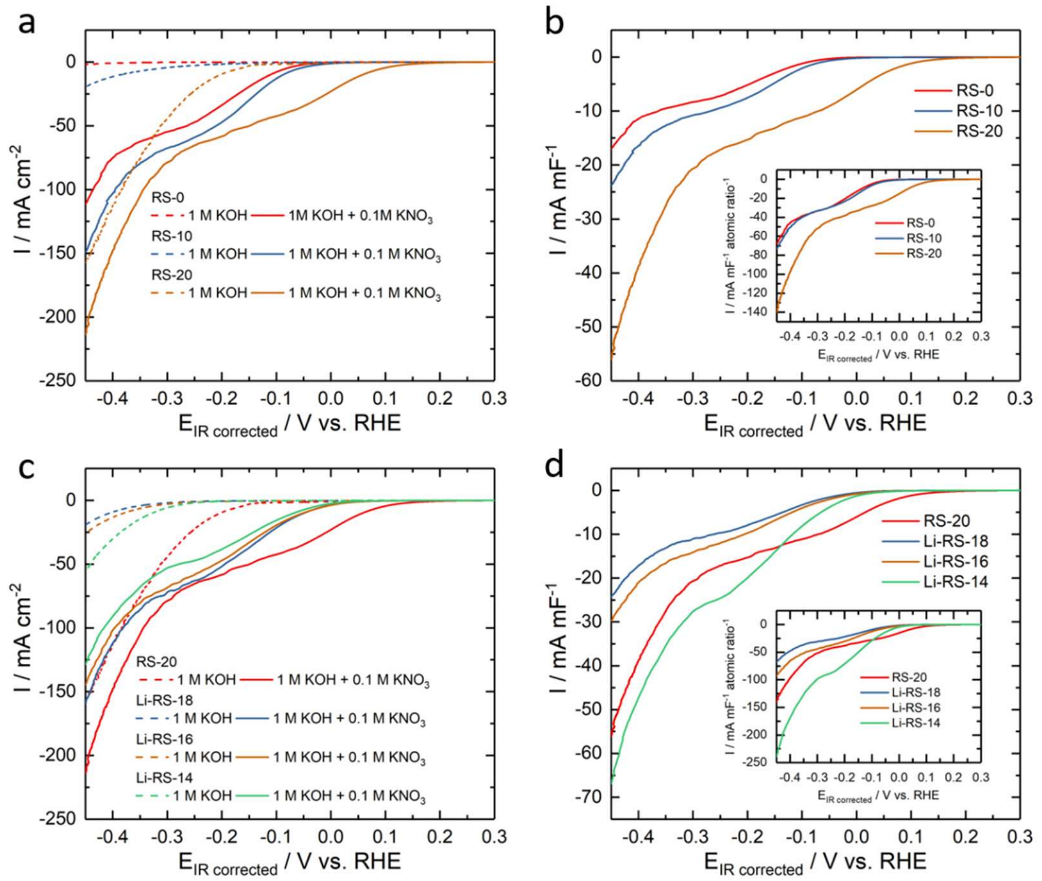

Supplementary Fig. 7 LSV curves of RS-0, RS-10, RS-20, Li-RS-18, Li-RS-16, and Li-RS-14. The increase of the Cu ratio in (**a**) and (**b**). The increase of the Li ratio in (**c**) and (**d**). The current density is normalized by glassy carbon's geometric area of 0.19625 cm^{-2} in (**a**) and (**c**). The current density is normalized by the electrochemical double-layer capacitance and Co + Cu atomic ratios (inset) in (**b**) and (**d**). Experimental condition: The mass loading of 1 mg_{oxide} cm^{-2} on the glassy carbon substrate (geometry area 0.19625 cm^2) for LSV tests in Ar-saturated 1 M KOH with or without 0.1 M KNO_3 at a scan rate of 10 mV s^{-1} with 85% IR correction. The system resistance of the electrochemical cell containing 1 M KOH with and without 0.1 M KNO_3 is as follow. RS-0, 4.635 ± 0.493 Ohm and 4.367 ± 0.523 Ohm. RS-10, 4.487 ± 0.221 Ohm and 3.674 ± 0.311 Ohm. RS-20, 6.26 ± 0.230 Ohm and 5.812 ± 0.138 Ohm. Li-RS-18, 5.74 ± 0.667 Ω Ohm and 5.115 ± 0.189 Ohm. Li-RS-16, 5.802 ± 0.135 Ohm and 4.293 ± 0.732 Ohm. Li-RS-14, 4.528 ± 0.032 Ohm and 4.635 ± 0.162 Ohm.

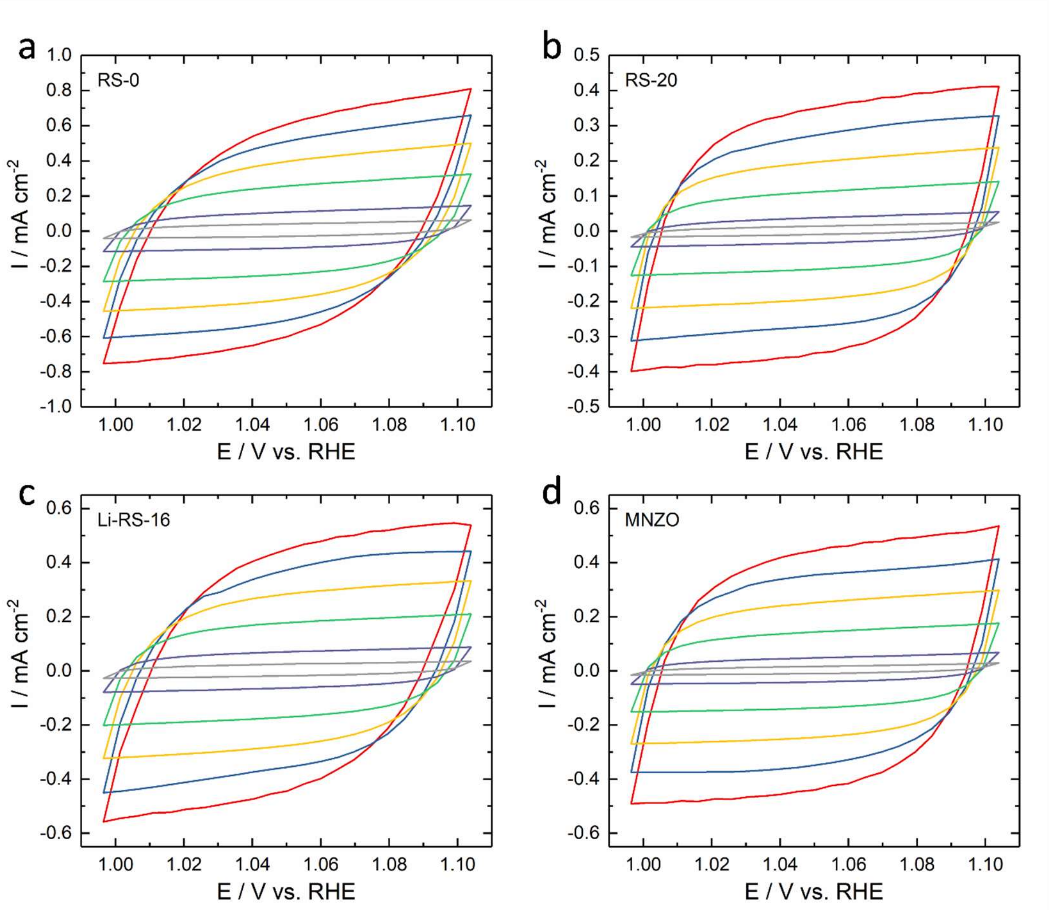

Supplementary Fig. 8 CV curves of (**a**) RS-0, (**b**) RS-20, (**c**) Li-RS-16 and (**d**) MNZO to obtain double-layer capacitances at scan rates of 100, 80, 60, 40, 20 and 10 mV s^{-1} in 1 M KOH. The geometry area of the working electrode (glassy carbon) was 0.19625 cm^2, and the catalyst loading was 1 mg_{oxide} cm^{-2}. The system resistance of the electrochemical cell can be found in Supplementary Fig. 7.

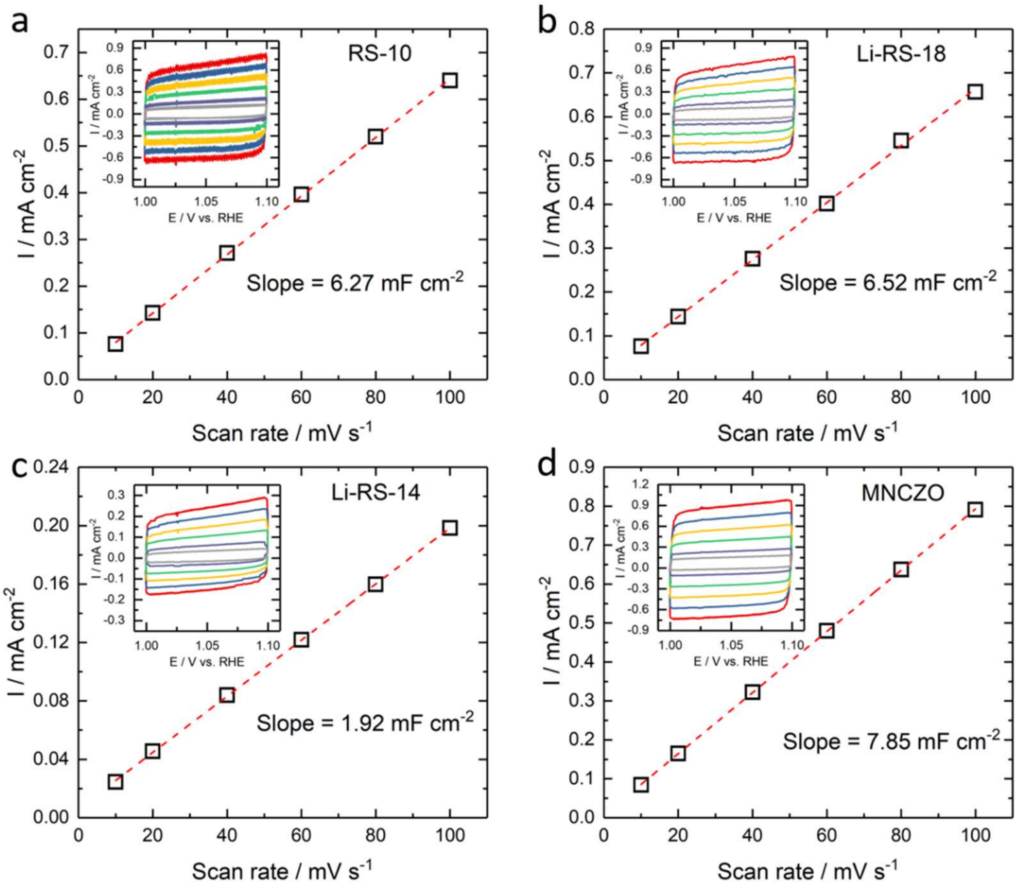

Supplementary Fig. 9 CV curves in 1 M KOH and current density-scan rate linear fittings to obtain electrochemical double-layer capacitances: (**a**) RS-10, (**b**) Li-RS-18, (**c**) Li-RS-14 and (**d**) MNCZO. The geometry area of the working electrode (glassy carbon) was 0.19625 cm^2, and the catalyst loading was 1 mg_{oxide} cm^{-2}. The system resistance of the electrochemical cell can be found in Supplementary Fig. 7.

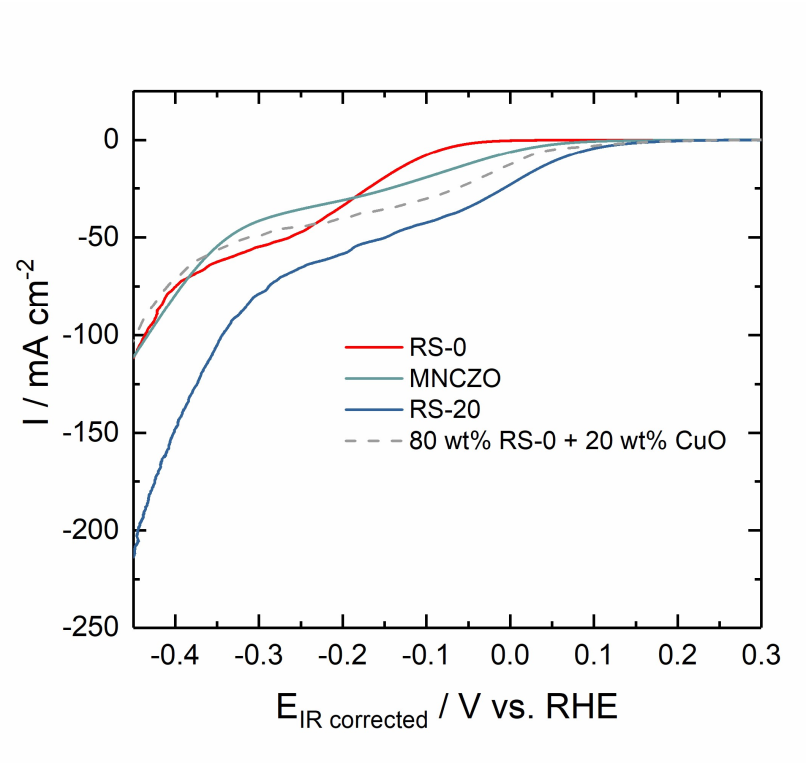

Supplementary Fig. 10 LSV curves of RS-0, $Mg_{0.25}Ni_{0.25}Cu_{0.25}Zn_{0.25}O$ (MNCZO), RS-20, and the mixture of 80 wt% RS-0 and 20 wt% CuO in 1 M KOH + 0.1 M KNO_3 solution at a scan rate of 10 mV s^{-1} with 85% IR correction. The geometry area of the working electrode (glassy carbon) was 0.19625 cm^2, and the catalyst loading was 1 mg_{oxide} cm^{-2}. The system resistance of the electrochemical cell containing 1 M KOH and 0.1 M KNO_3 is as follow. RS-0, 4.635 ± 0.493 Ohm. MNCZO, 3.718 ± 0.088 Ohm. RS-20, 5.812 ± 0.138 Ohm; RS-0 & CuO, 6.200 ± 0.263 Ohm.

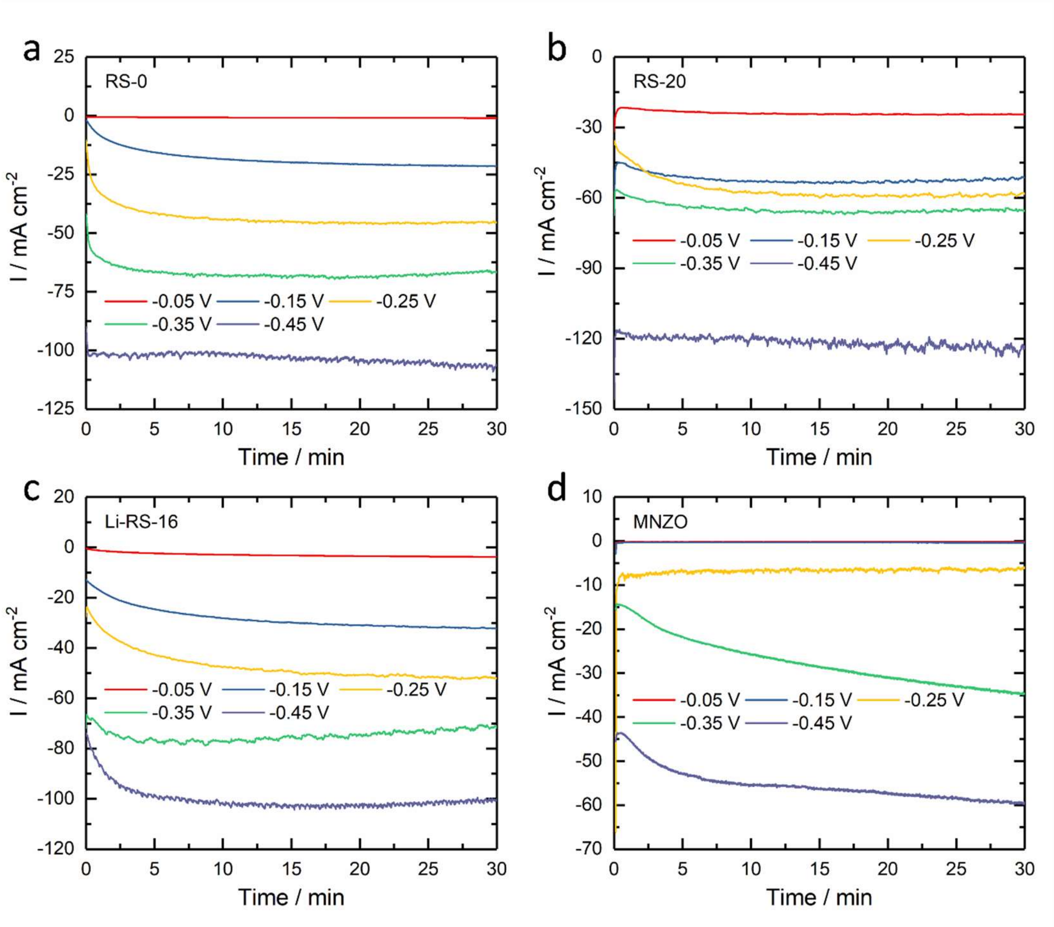

Supplementary Fig. 11 Chronoamperometry curves in 1 M KOH + 0.1 M KNO_3. a RS-0. **b** RS-20. **c** Li-RS-16. **d** MNZO. Experimental condition: The mass loading of 1 mg_{oxide} cm^{-2} on the Toray 090 carbon paper (1.5 × 1.5 cm^2) for nitrate electrolysis at the designed potential for 30 min in a H-cell with FAA-3-PK-130 anion exchange membrane (FuMA-Tech) separating two chambers. Each chamber had 18 mL of 1 M KOH and 0.1 M KNO_3 electrolyte saturated with Ar. The system resistance of the electrochemical cell containing 1 M KOH and 0.1 M KNO_3 is as follow. RS-0, 0.861 ± 0.112 Ohm. RS-20, 0.828 ± 0.074 Ohm. Li-RS-16, 0.802 ± 0.130 Ohm. MNZO,0.896 ± 0.107 Ohm.

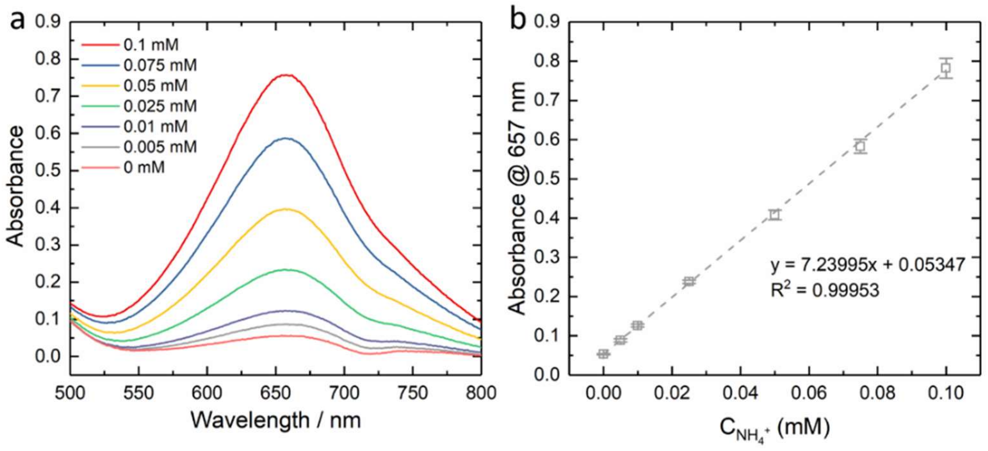

Supplementary Fig. 12 ammonia quantification. **a** UV-vis spectra. **b** absorbance-NH_4^+ concentration fitting.

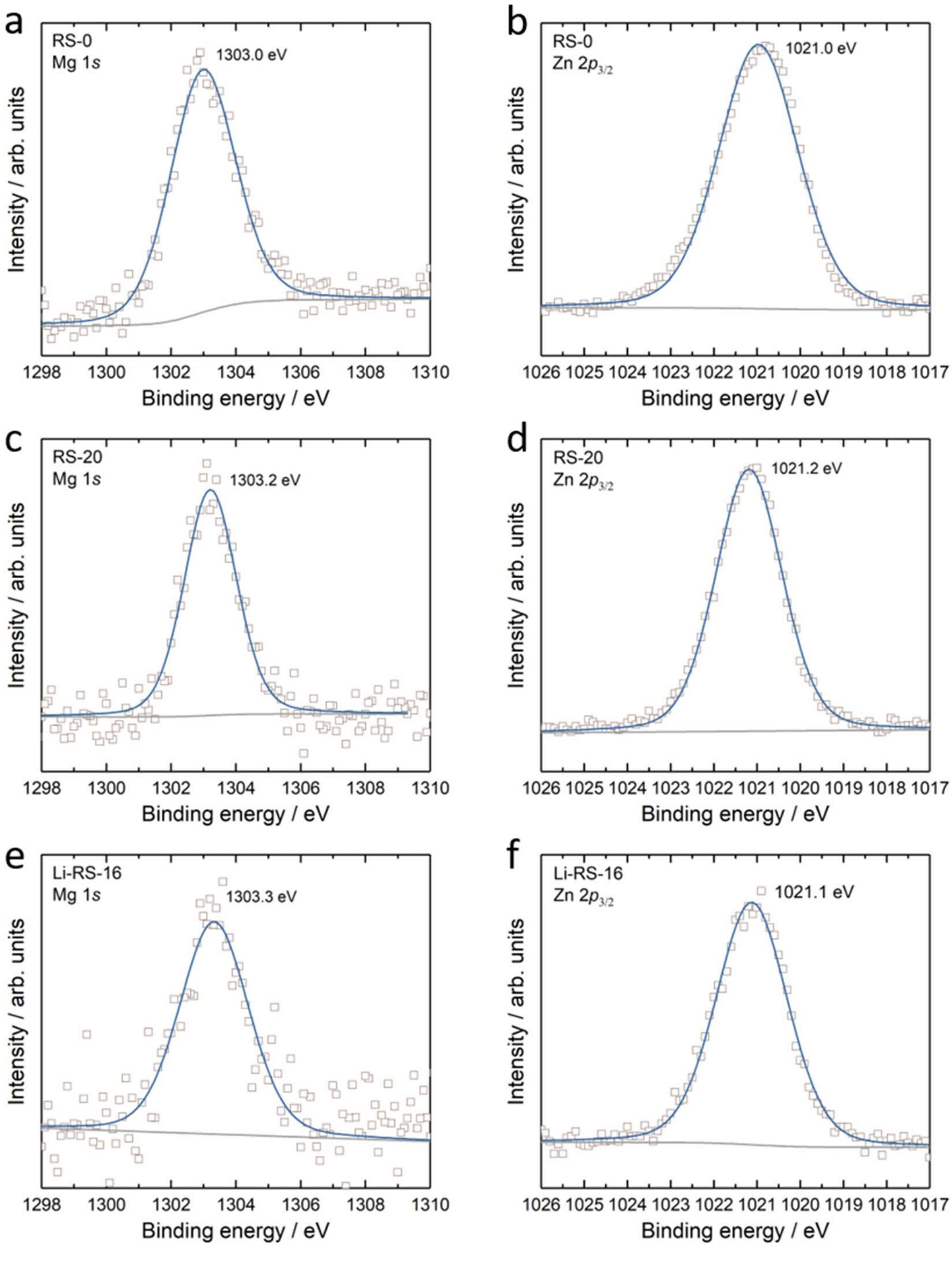

Supplementary Fig. 13 Mg 1*s* and Zn 2$p_{3/2}$ XPS curves of (**a-b**) RS-0, (**c-d**) RS-20 and (**e-f**) Li-RS-16.

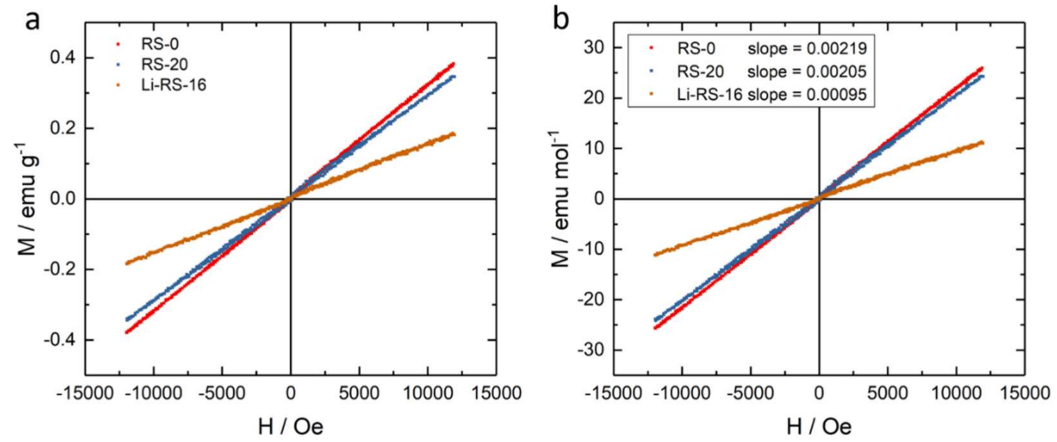

Supplementary Fig. 14 The M-H curves of RS-0, RS-20, and Li-RS-16 at room temperature. **a** Magnetic moment normalized by mass. **b** Magnetic moment normalized by mole number.

We conducted the VSM test at room temperature using Lake Shore 7400 VSM. Supplementary Fig. 14 shows the M-H curves of RS-0, RS-20 and Li-RS-16, where **M** is the magnetization, **H** is the magnetic field strength. The magnetic susceptibility χ can be obtained based on the equation $\mathbf{M} = \chi\mathbf{H}$. The linear M-H relationship and the positive χ values of RS-0, RS-20, Li-RS-16 indicate that these three samples are paramagnetic at room temperature. From the relationship $\chi = \mathrm{N}g^2 J(J+1)\mu_B^2 / (3k_B T)$, where N, g, J, μ_B, k_B and T are the Avogadro number, g-factor, angular momentum quantum number, Bohr magneton, Boltzmann constant and temperature, respectively. A larger χ value means a larger J, indicating a higher spin state. The M-H curves shows that RS-0 and RS-20 have a very close magnetic susceptibility. Li-RS-16 has a smaller magnetic moment than RS-0 and RS-20. The total mole fractions of Co, Ni and Cu in RS-0, RS-20 and Li-RS-16 are 0.50, 0.60 and 0.48, respectively. If the magnetic moment contribution is considered to merely from the Co, Ni and Cu, the magnetic moments of RS-0 and RS-20 are still higher than that of Li-RS-16. However, as the electronic states of Ni and Cu are not observed remarkably different, the magnetic moment decreases could come from the decrease in Co spin state.

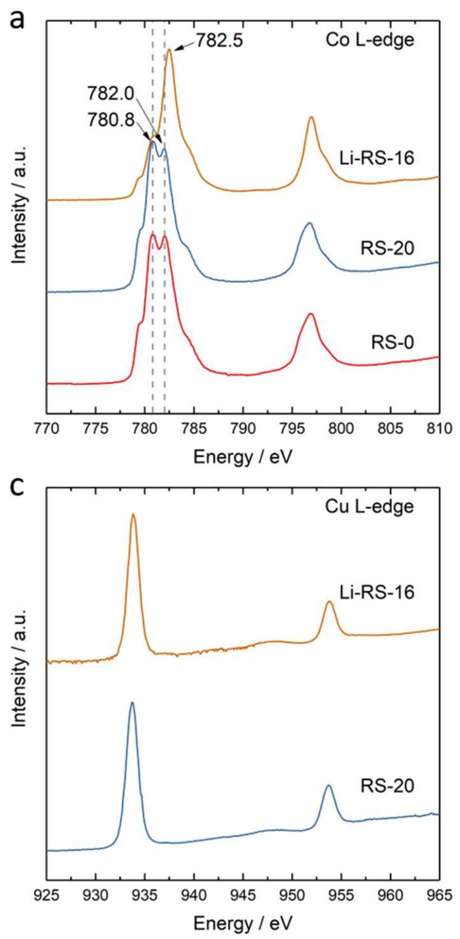

Supplementary Fig. 15 XAS of RS-0, RS-20, and Li-RS-16. a Co L-edge. **b** Ni L-edge. **c** Cu L-edge. **d** O K-edge.

Apart from VSM, we also used soft X-ray to characterize the Co, Ni and Cu L-edges and O K-edge of RS-0, RS-20, and Li-RS-16 (Supplementary Fig. 15). Soft X-ray absorption experiment was performed at the SUV beamline of Singapore Synchrotron Light Source (SSLS). The $L_{2,3}$-edges of Co, Ni, and Cu represent the transition from $2p^6 3d^n$ to $2p^5 3d^{n+1}$, where n is the electron number in the d orbitals. Take Co as an example, lower energy L_3 peak stands for the transition $2p_{3/2} \rightarrow 3d$ and higher energy L_2 peak stands for the transition $2p_{1/2} \rightarrow 3d^1$. The Co L_3 peak of RS-20 and RS-20 splits into two peaks at 780.8 eV and 782.0 eV with similar weight. The Co L_3 peak of Li-RS-16 and RS-20 splits into a weak peak at 780.8 eV and a strong peak 782.5 eV. Although the interpretation of Co L-edge requires the consideration of multiplet splitting, hybridization, and crystal field effects, here we simplify this behavior as energy splitting between the t_{2g} states and the e_g states[2]. In the O_h symmetry,

Co^{2+} has the low spin state $t_{2g}{}^6e_g{}^1$ and high spin state $t_{2g}{}^5e_g{}^2$, and Co^{3+} has the low spin state $t_{2g}{}^6e_g{}^0$, intermediate spin state $t_{2g}{}^5e_g{}^1$, and high spin state $t_{2g}{}^4e_g{}^2$. Therefore, the peak intensity theoretically corresponds to the hole number of t_{2g} (at low energy) and e_g (at high energy). The Co L_3-edges of RS-0 and RS-20 have no obvious change, indicating no electron occupation change. When comparing Li-RS-16 and RS-20, the peak corresponding to e_g orbitals shifts to the high energy direction relative to RS-20. This indicates that the Co valence state increases, in agreement with our XANES results and the previous investigation[3]. In Li-RS-16, the peak corresponding to e_g is much stronger than the peak corresponding to t_{2g}, indicating that there are less holes in t_{2g} orbitals and more holes in e_g orbitals. For Co^{2+} or Co^{3+}, it suggests there are less unpaired electrons and exhibits lower spin. Besides, there is a larger peak splitting energy between t_{2g} and e_g orbitals in Li-RS-16 than in RS-0 and RS-20, which is consistent with EXAFS results that Co-O distance is smaller in Li-RS-16 than in RS-0 and RS-20. A smaller Co-O causes a strong crystal field that induces a larger t_{2g} and e_g splitting.

For Ni and Cu cations, we did not observe the obvious difference among RS-0, RS-20 and Li-RS-16. Since the O K-edge represents the electronic transitions from the O 1*s* core level to the unoccupied transition metal 3*d* levels with the O 2*p* components, due to the coexistence of multiple cations, we cannot use it to analyse the d-orbital occupation of cations. A remarkable difference is that the signal reflecting the hole in oxygen (at ~531.5 eV) becomes stronger from RS-0, RS-20 to Li-RS-16, which is consistent with our EELS results. Another difference is the disappearance of the peak at 533.2 eV representing the metal-oxygen covalency[4], which could be due to the highly ionic Li-O bond.

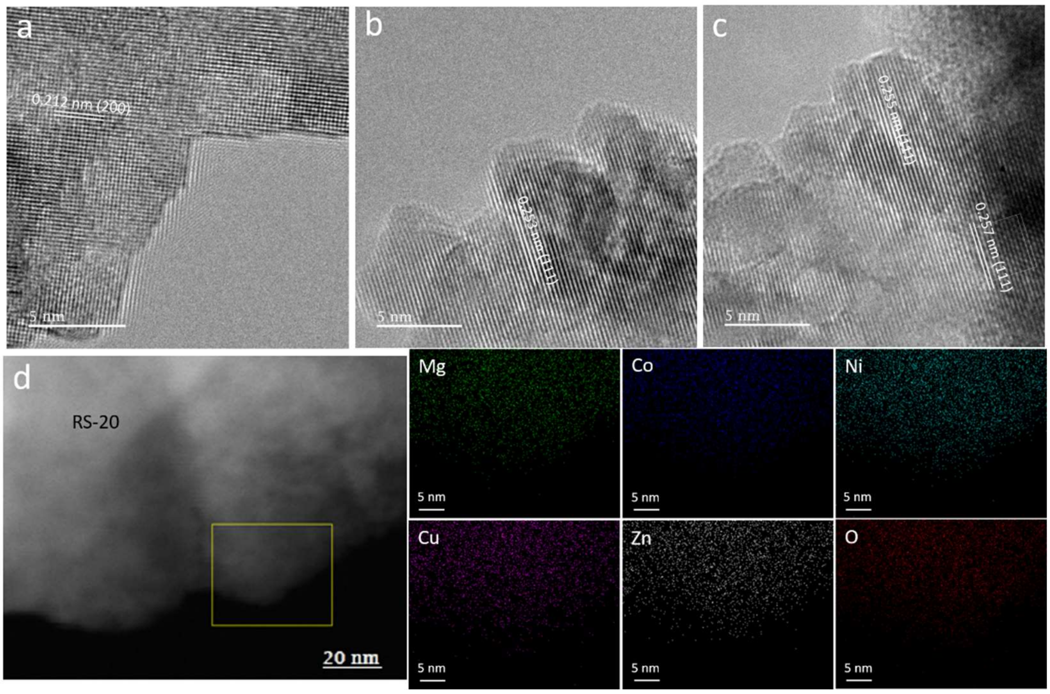

Supplementary Fig. 16 HRTEM images (**a-c**) and TEM-EDX mapping (**d**) of RS-20 after nitrate reduction at -0.35 V for 30 min.

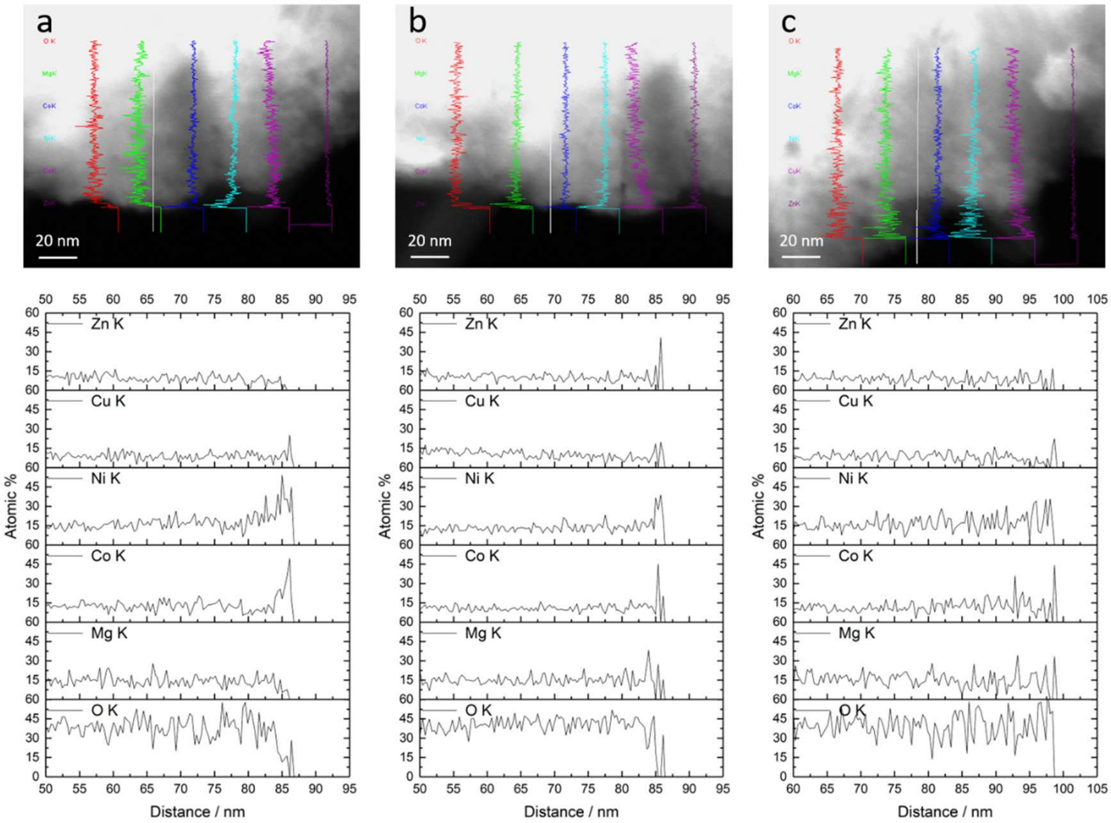

Supplementary Fig. 17 TEM-EDX line scans of RS-20 after nitrate reduction at -0.35 V for 30 min, (**a-c**) indicating the different regions.

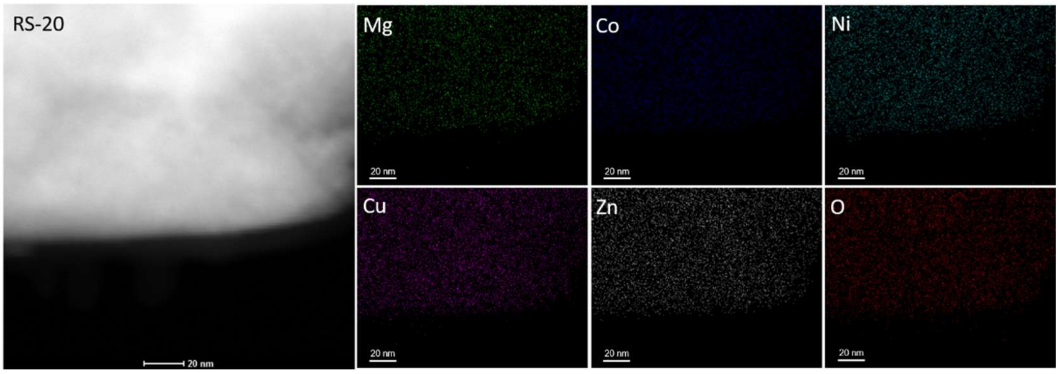

Supplementary Fig. 18 TEM-EDX mapping of RS-20 prior to nitrate reduction.

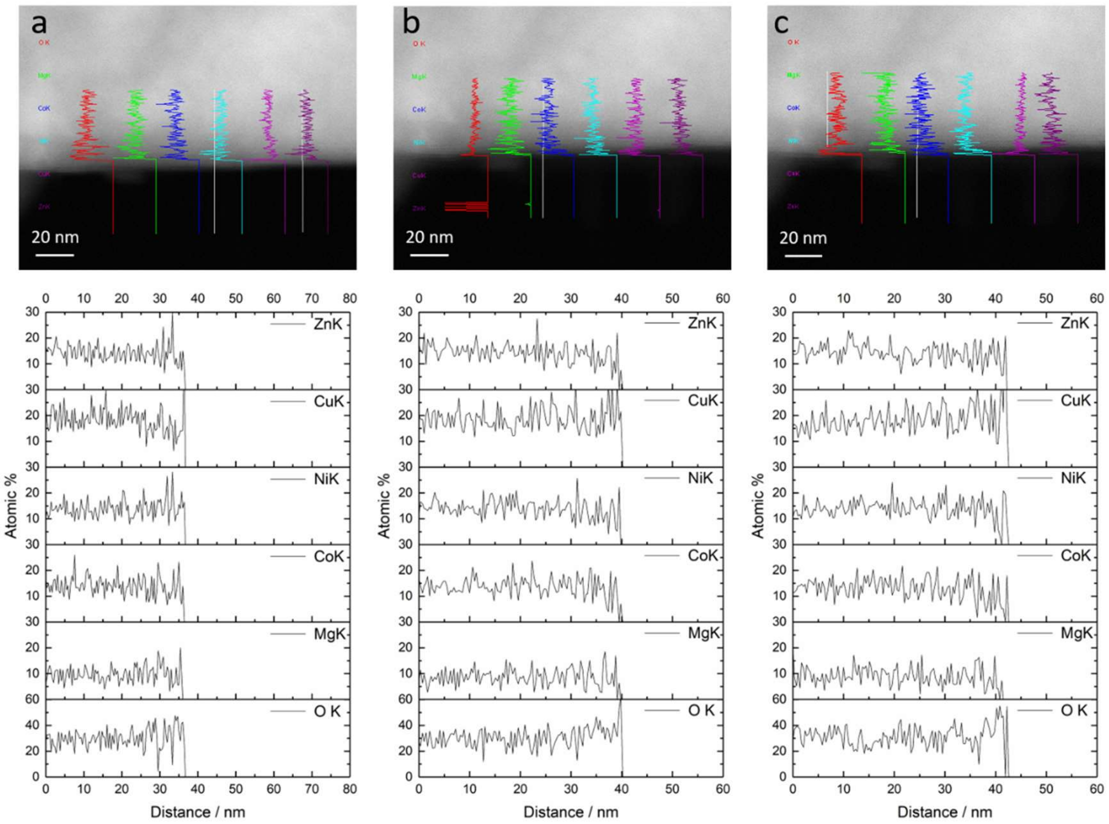

Supplementary Fig. 19 TEM-EDX line scans of RS-20 prior to nitrate reduction, (**a-c**) indicating the different regions.

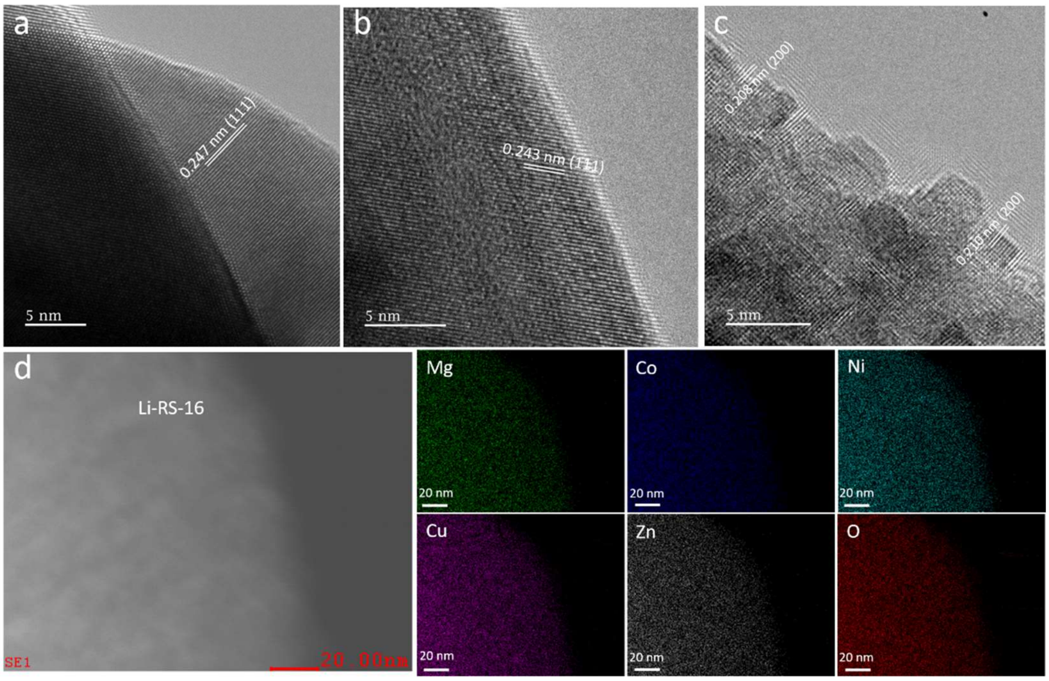

Supplementary Fig. 20 HRTEM images (**a-c**) and TEM-EDX mapping (**d**) of Li-RS-16 after nitrate reduction at -0.35 V for 30 min.

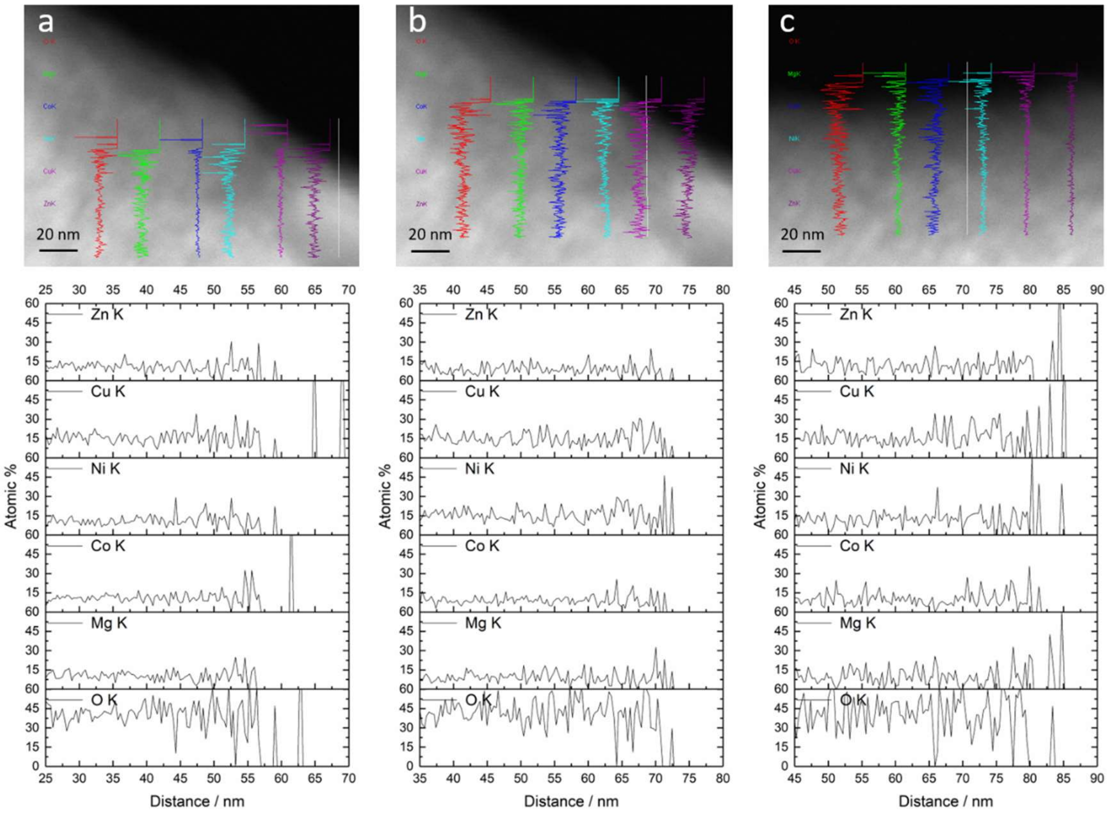

Supplementary Fig. 21 TEM-EDX line scan of Li-RS-16 after nitrate reduction at -0.35 V for 30 min, (**a-c**) indicating the different regions.

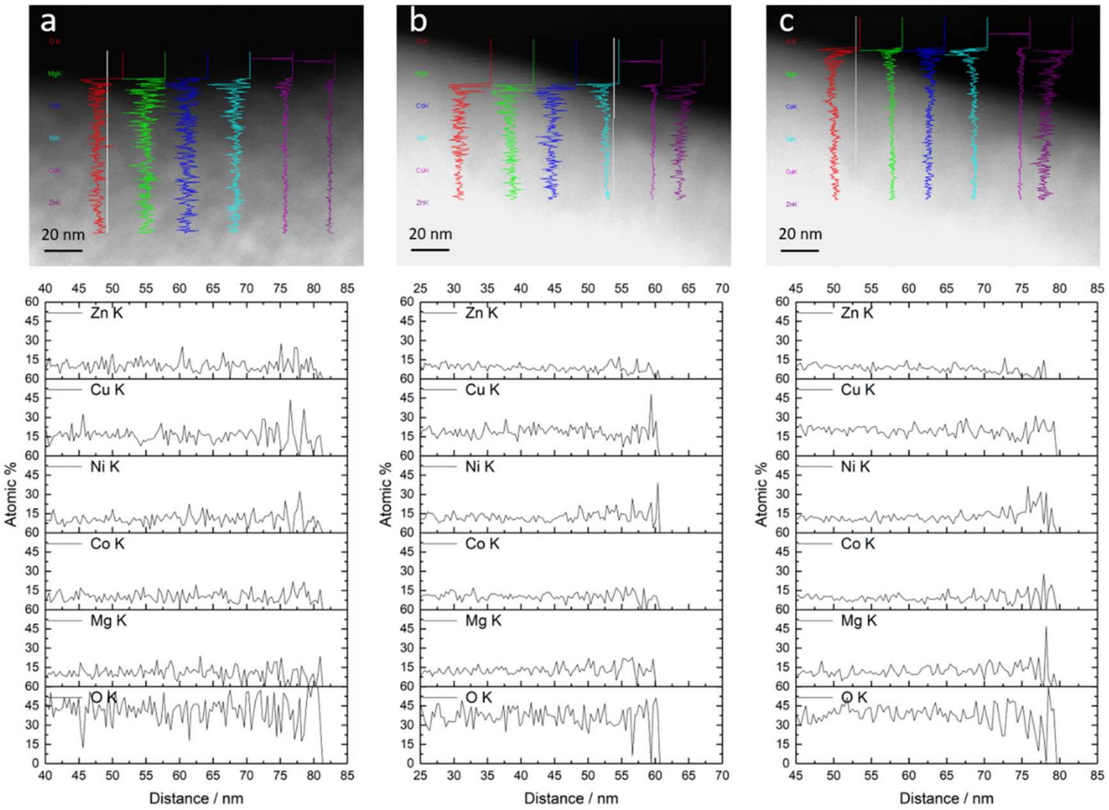

Supplementary Fig. 22 TEM-EDX line scan of Li-RS-16 after nitrate reduction at -0.35 V for 30 min, (**a-c**) indicating the different regions.

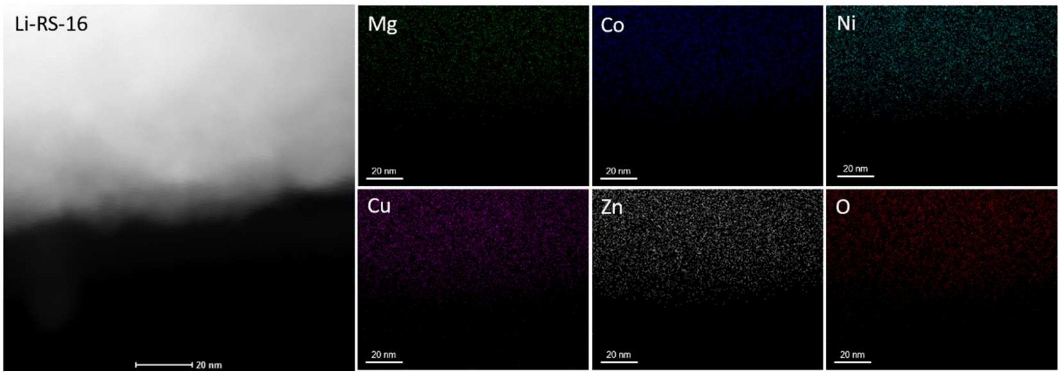

Supplementary Fig. 23 TEM-EDX mapping of Li-RS-16 prior to nitrate reduction.

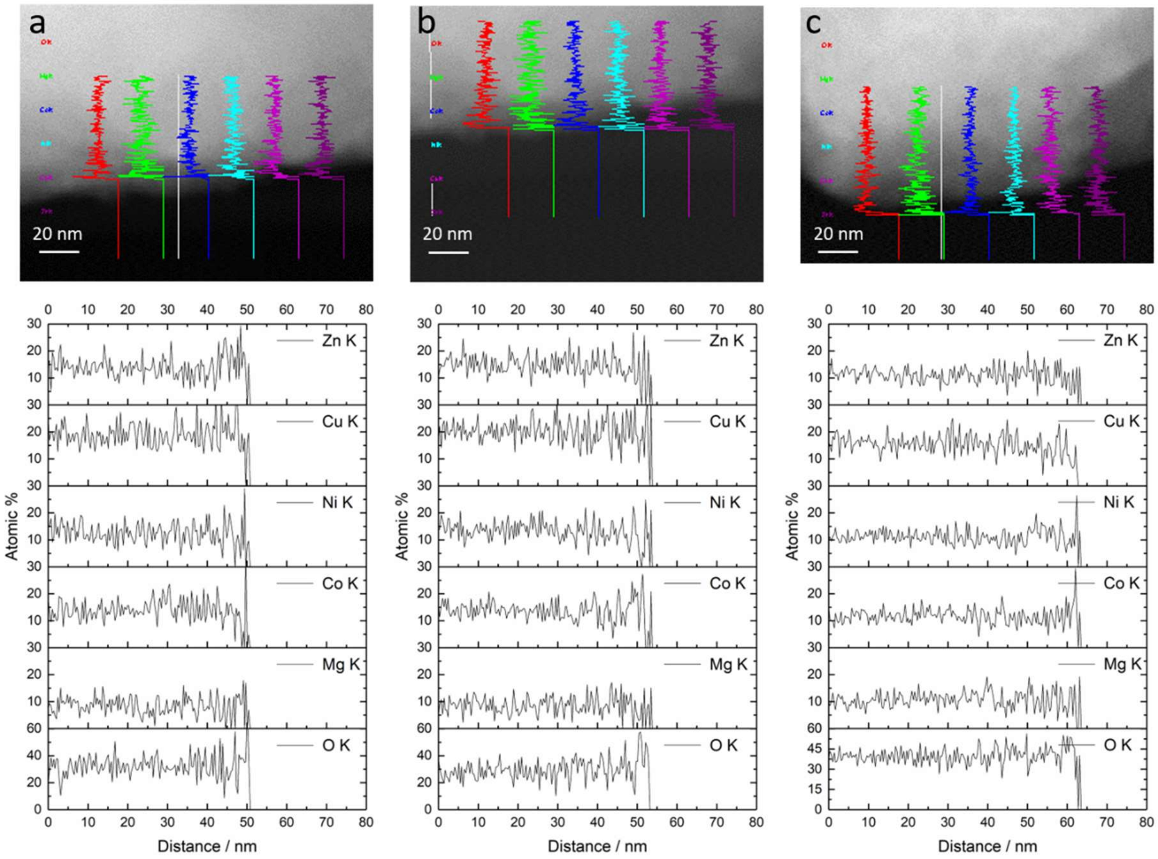

Supplementary Fig. 24 TEM-EDX line scans of Li-RS-16 prior to nitrate reduction, (**a-c**) indicating the different regions.

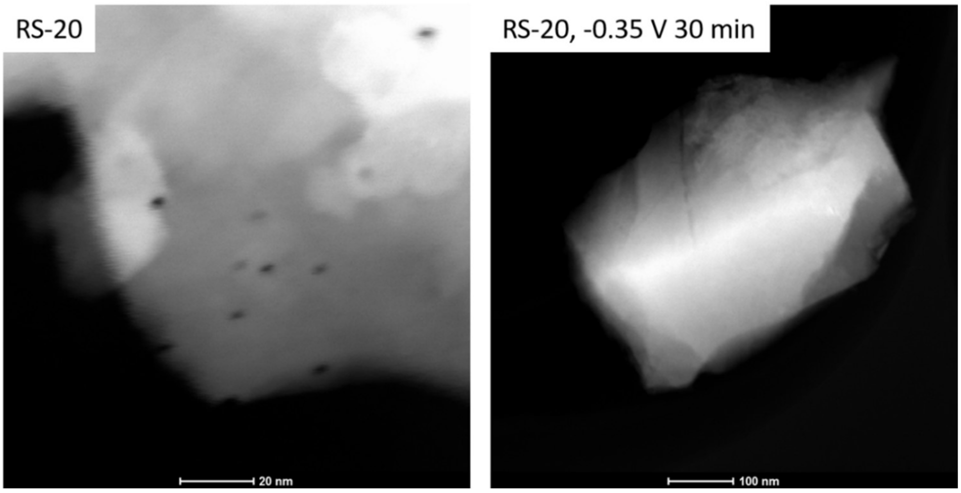

Supplementary Fig. 25 High-angle annular dark-field scanning transmission electron microscopy (HAADF-STEM) images for EELS measurements of RS-20 prior to and after nitrate reduction at -0.35 V for 30 min in 1 M KOH + 0.1 M KNO_3.

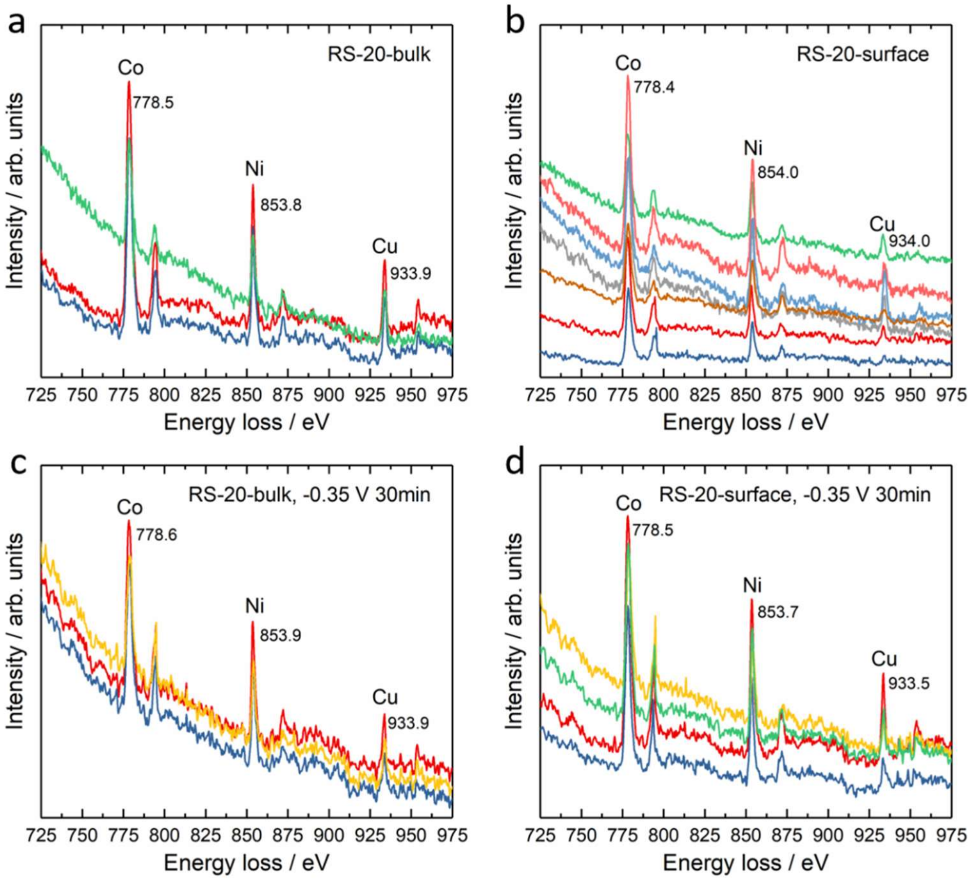

Supplementary Fig. 26 Co, Ni and Cu EELS measurements for the bulk and surface of RS-20 prior to and after nitrate reduction at -0.35 V for 30 min in 1 M KOH + 0.1 M KNO_3. **a** Bulk prior to nitrate reduction. **b** Surface prior to nitrate reduction. **c** Bulk after nitrate reduction. **d** Surface after nitrate reduction. All data are aligned with oxygen peaks in Fig. 2f.

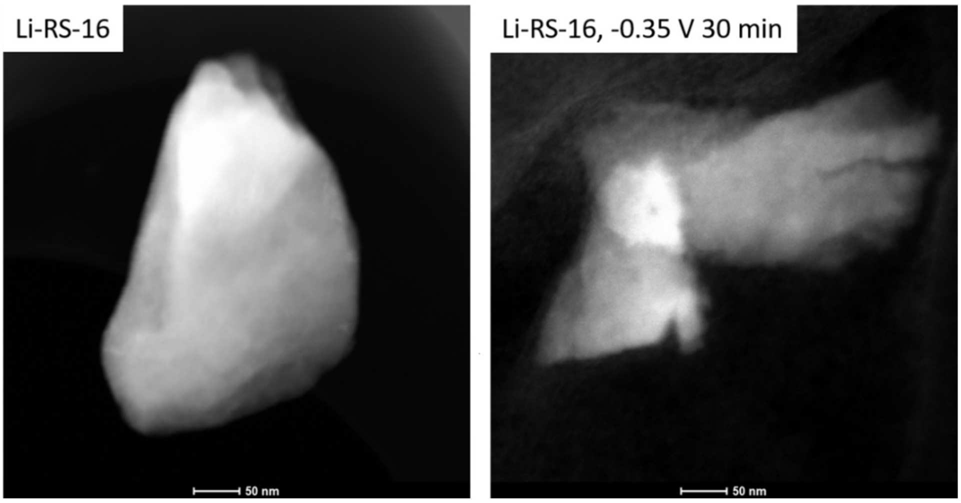

Supplementary Fig. 27. HAADF-STEM images for EELS measurements of Li-RS-16 prior to and after nitrate reduction at -0.35 V for 30 min in 1 M KOH + 0.1 M KNO_3.

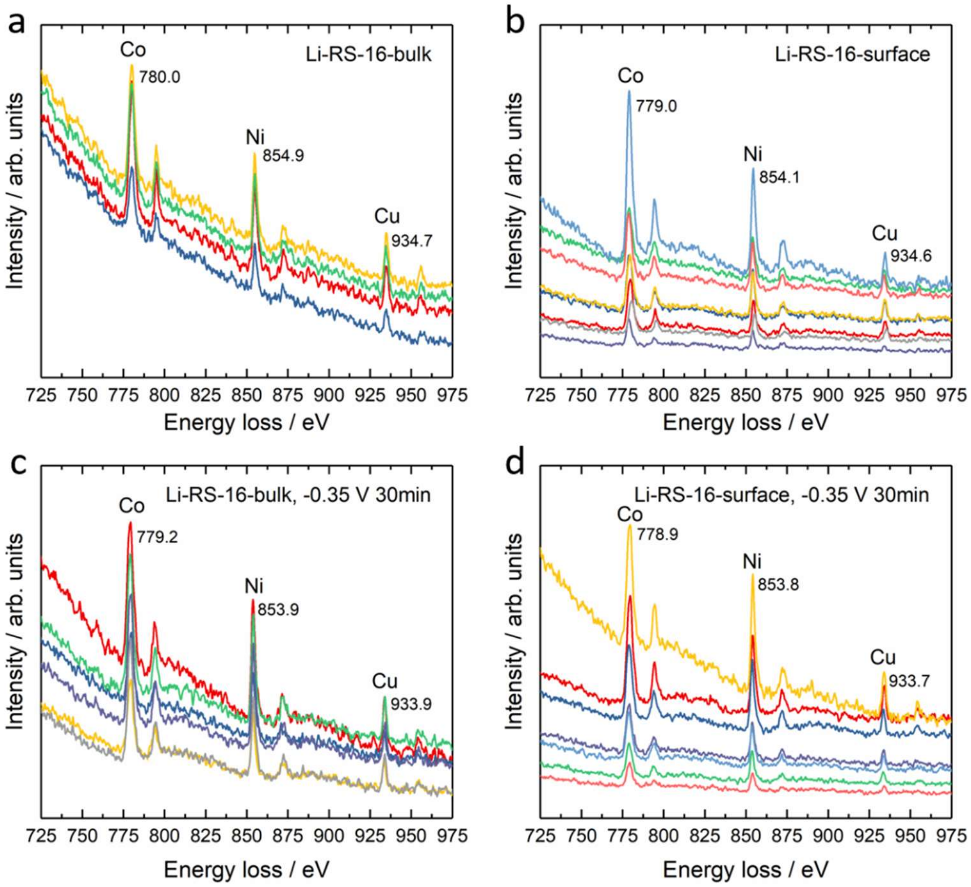

Supplementary Fig. 28 Co, Ni and Cu EELS measurements for the bulk and surface of Li-RS-16 prior to and after nitrate reduction at -0.35 V for 30 min in 1 M KOH + 0.1 M KNO_3. a Bulk prior to nitrate reduction. **b** Surface prior to nitrate reduction. **c** Bulk after nitrate reduction. **d** Surface after nitrate reduction. All data are aligned with oxygen peaks in Fig. 2f.

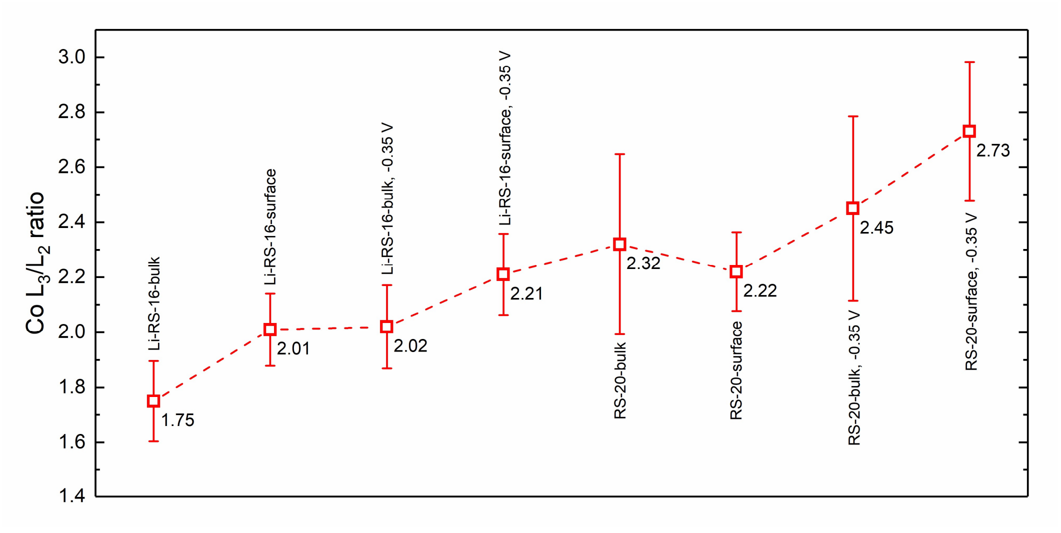

Supplementary Fig. 29 Co L_3/L_2 ratios from EELS measurements for the bulk and surface of RS-20 and Li-RS-16 prior to and after nitrate reduction at -0.35 V for 30 min in 1 M KOH + 0.1 M KNO_3. The standard deviations come from at least three independent measurements.

We used EELS to probe the electronic states of Co and Ni and Cu at different locations of the bulk and surface before and after nitrate reduction (Supplementary Figs. 25-28). Statistical results are given in Supplementary Fig. 29. The change of Co L_3/L_2 intensity ratio can describe the change in Co valence states[5]. Smaller ratios represent higher valence states. After nitrate reduction, Co L_3/L_2 ratios in RS-20 and Li-RS-16 increase at the surface and bulk, with the ratios at the surface bigger than the bulk. It is consistent with XANES results that Co can be reduced after reduction (Fig. 3). The Co valence state trend of RS-20 and Li-RS-16 from EELS measurement is agreement with our XANES results. Moreover, the bigger L_3/L_2 ratio at the surface indicates a bigger reduction degree. For Ni, the intensity between Ni L_3 and L_2 peaks can be used to evaluate the Ni valence state, and a higher intensity after the L_3 peak represents a lower valence state[6]. For Cu, the location of the Cu L_3 peak can be used to evaluate the Cu valence state, and the peak location in the higher energy means a lower valence state[7]. In our EELS results, an obvious reduction of Ni and Cu can be observed, it can be explained through combining with XANES results that the reduction of Ni and Cu is slight (Fig. 3).

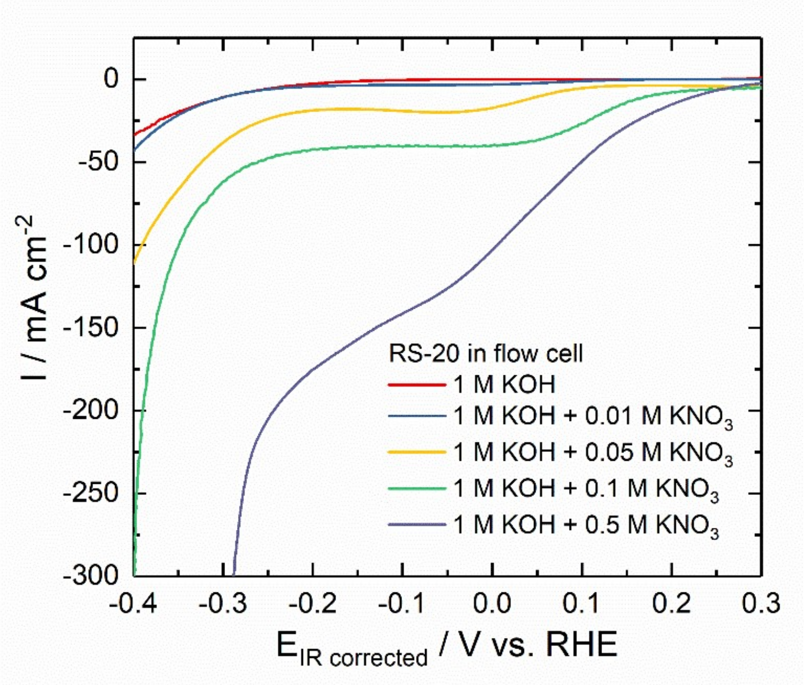

Supplementary Fig. 30 LSV curves of RS-20 in 1 M KOH in the absence of KNO_3 and in the presence of 0.01 M, 0.05 M, 0.1 M and 0.5 M KNO_3 at a scan rate of 10 mV s^{-1} with 85% IR correction in the flow cell. The area of the working electrode was 2 × 2 cm^2, and the catalyst loading was 0.5 mg_{oxide} cm^{-2}. The system resistance of the flow cell containing 1 M KOH and various concentrations of KNO_3 is as follow. No KNO_3, 0.381 ± 0.054 Ohm. 0.01 M KNO_3, 0.377 ± 0.021 Ohm. 0.05 M KNO_3, 0.343 ± 0.028 Ohm. 0.1 M KNO_3, 0.289 ± 0.012 Ohm. 0.5 M KNO_3, 0.295 ± 0.034 Ohm.

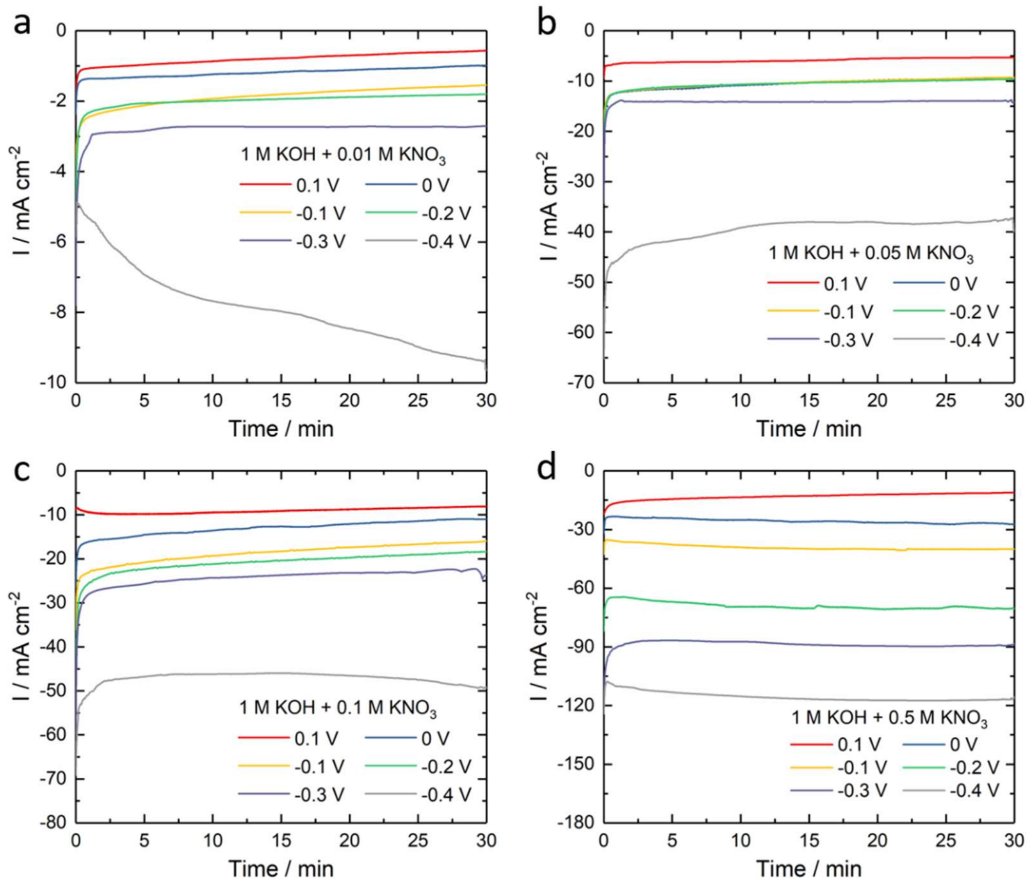

Supplementary Fig. 31 Chronoamperometry curves in the flow cell. RS-20 in (**a**) 1 M KOH + 0.01 M KNO_3, (**b**) 1 M KOH + 0.05 M KNO_3, (**c**) 1 M KOH + 0.1 M KNO_3, and (**d**) 1 M KOH + 0.1 M KNO_3. The area of the working electrode was 2 × 2 cm^2, and the catalyst loading was 0.5 mg_{oxide} cm^{-2}. The system resistance of the flow cell can be found in Supplementary Fig. 30.

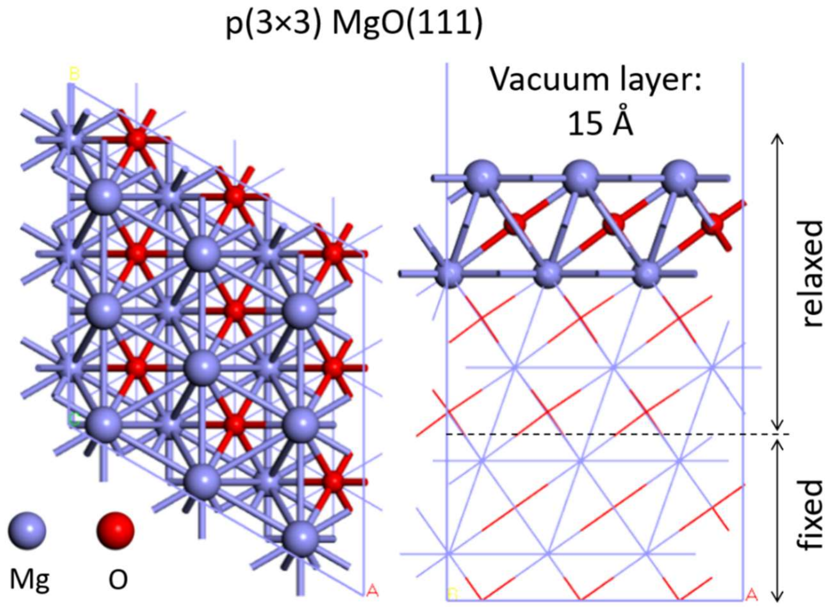

Supplementary Fig. 32 Top and side views of the MgO (111) surface model.

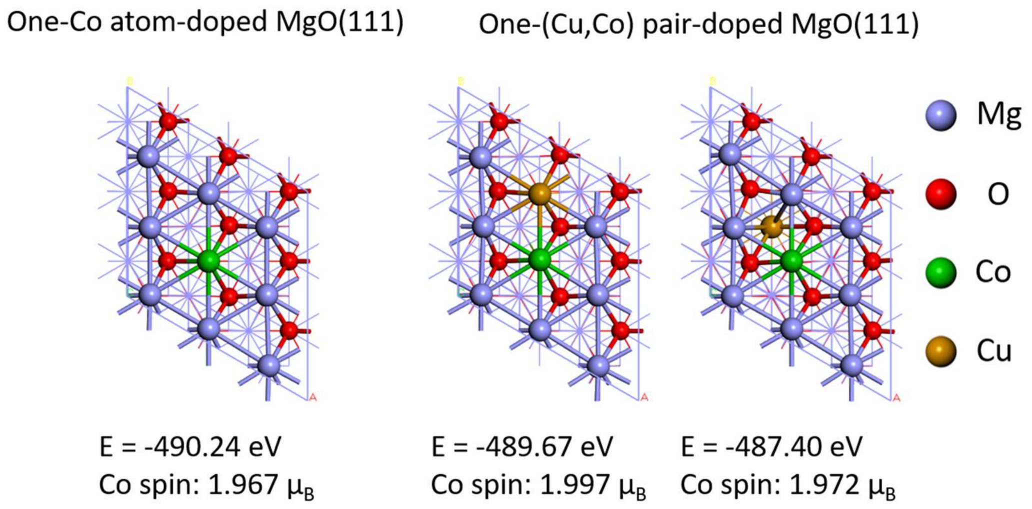

Supplementary Fig. 33 The possible cases of the energy and Co spin in the one-Co atom-doped MgO(111) and one-(Cu,Co) pair-doped MgO(111) surfaces.

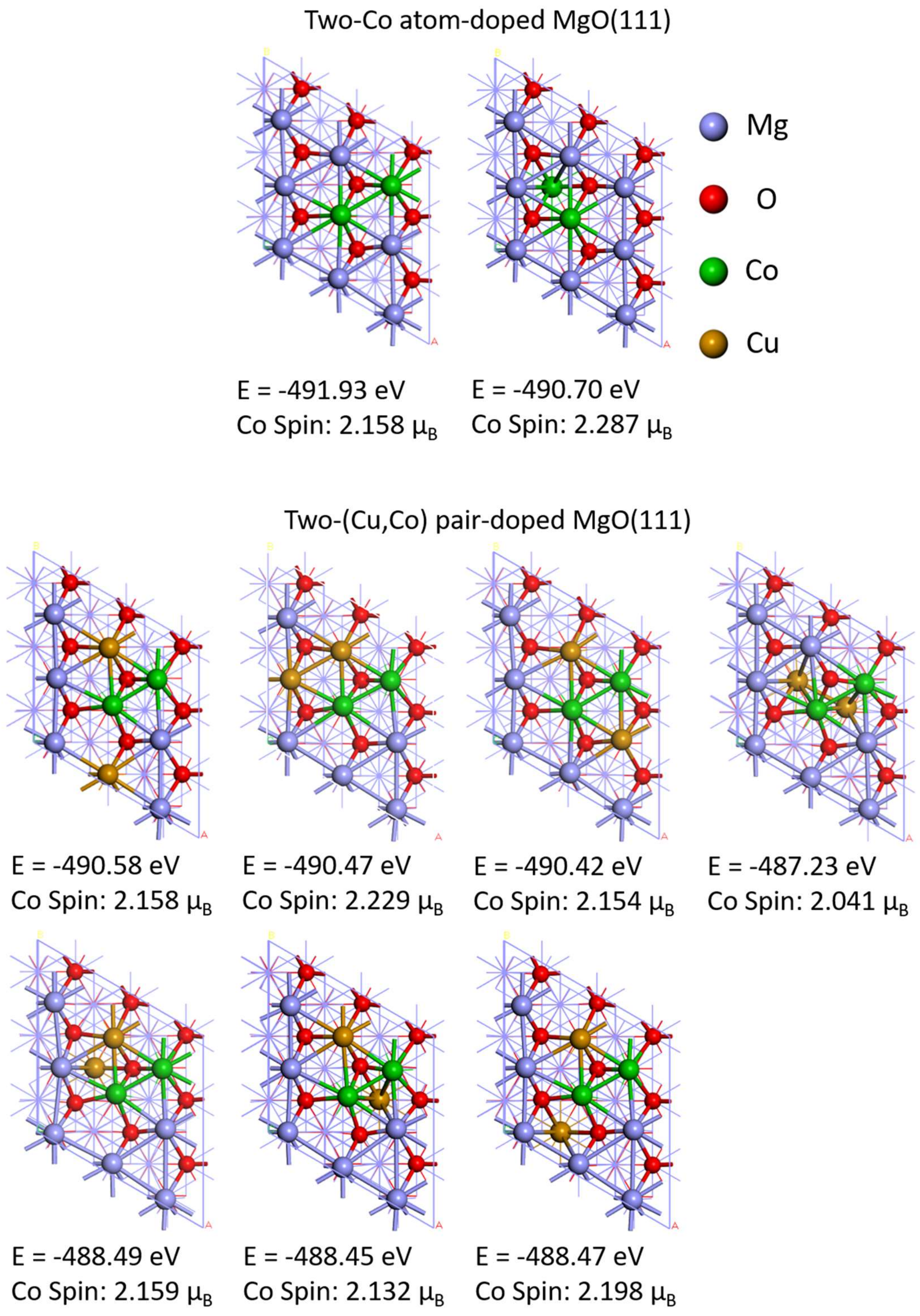

Supplementary Fig. 34 The possible cases of the energy and average Co spin in the two-Co atom-doped MgO(111) and two-(Cu,Co) pair-doped MgO(111) surfaces.

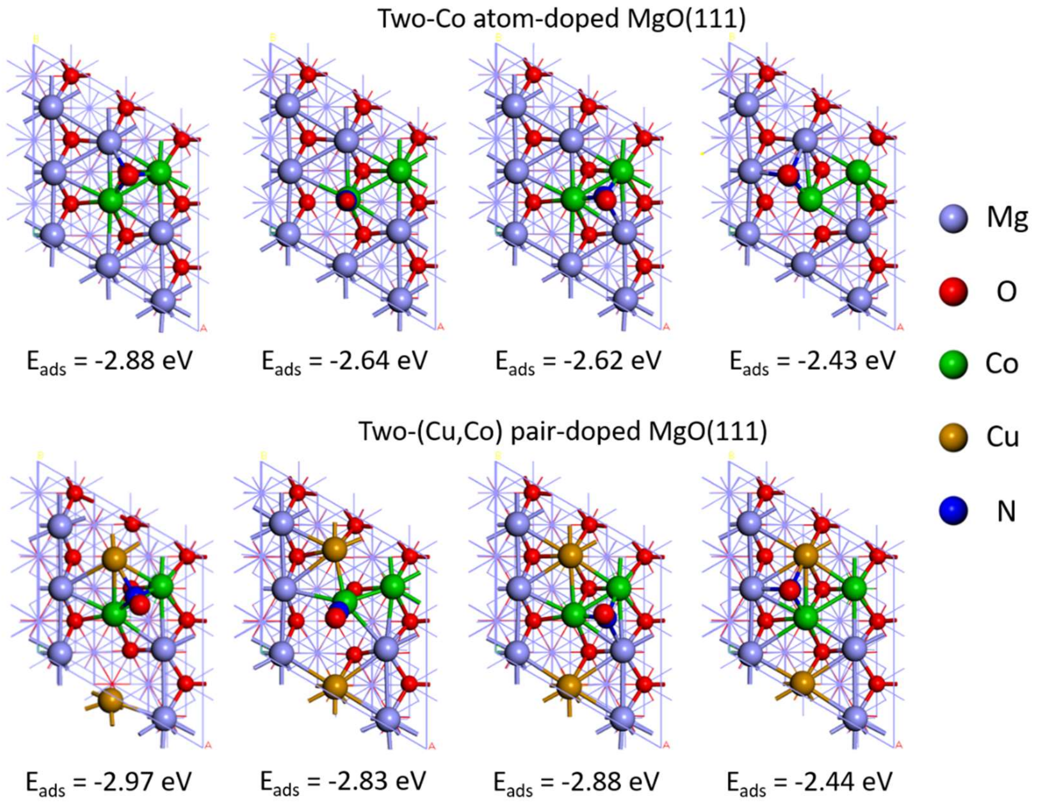

Supplementary Fig. 35 Possible configurations and adsorption energies of NO adsorption on the two-Co atom-doped MgO(111) and two-(Cu,Co) pair-doped MgO(111) surfaces.

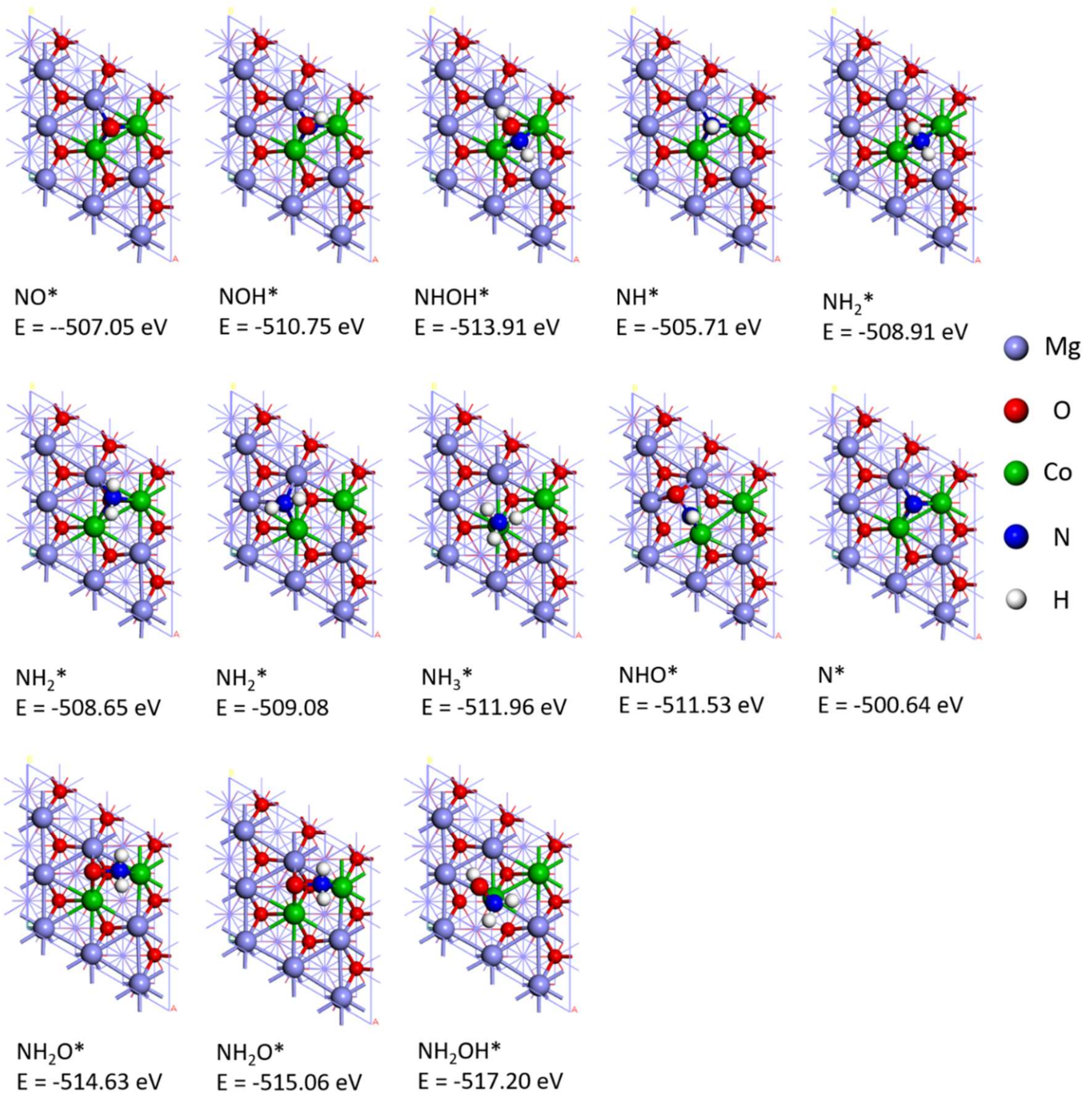

Supplementary Fig. 36 All possible intermediates during NO reduction to NH_3 on the two-atom Co-doped MgO(111) surfaces.

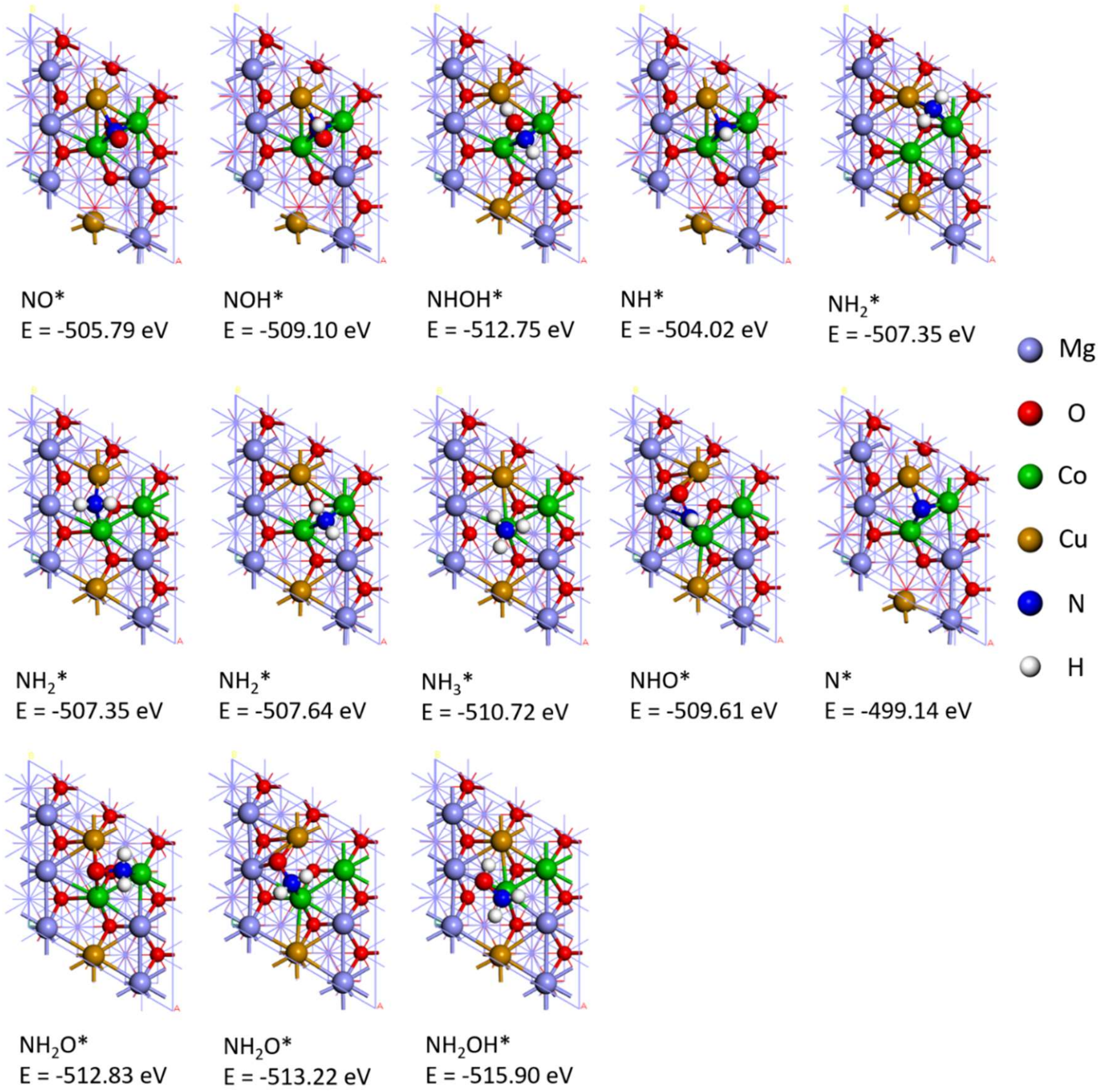

Supplementary Fig. 37 All possible intermediates during NO reduction to NH_3 on the two-(Cu,Co) pair-doped MgO(111) surfaces.

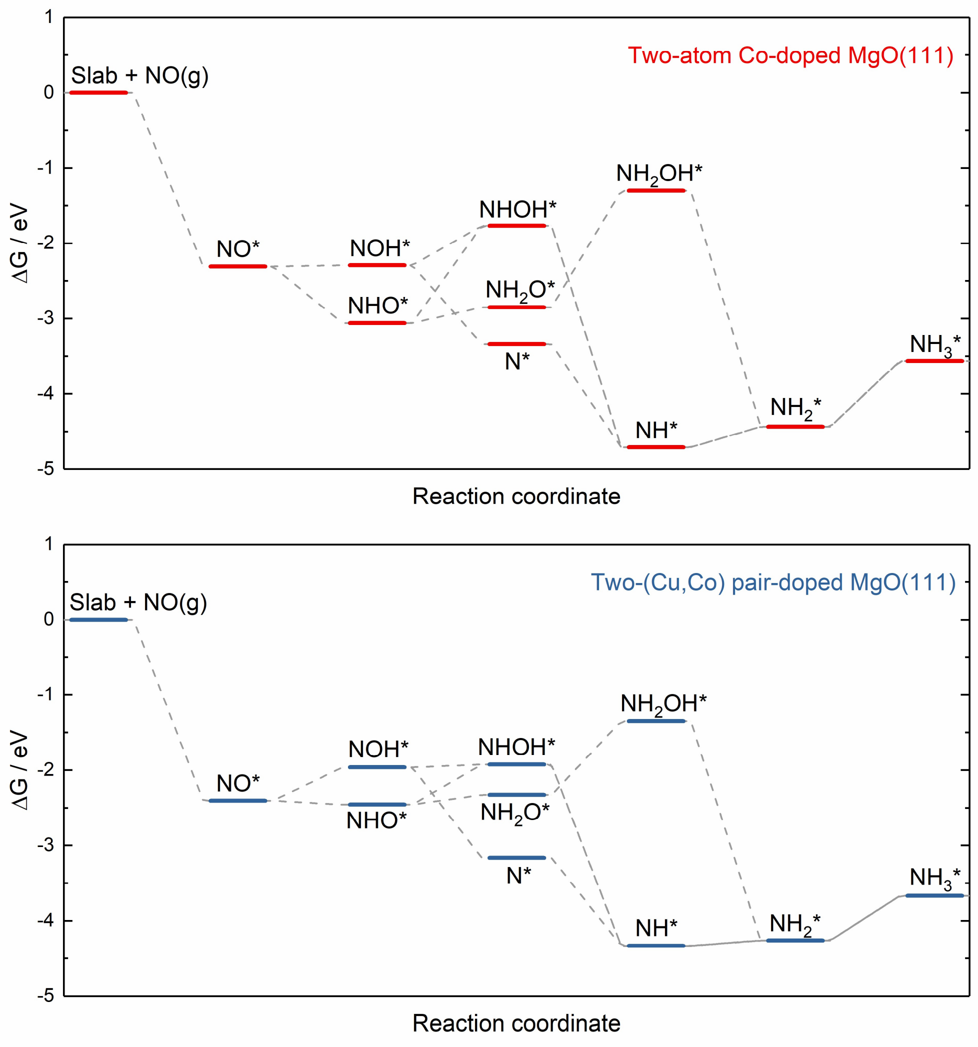

Supplementary Fig. 38 The calculated free energy diagram for the possible pathways of NO reduction to NH_3 on the two-atom Co-doped MgO(111) and two-(Cu,Co) pair-doped MgO(111) surfaces (298.15 K, 1 atmosphere).

Oxide stability

Stability is important factor when considering the reconstruction after electrolysis. Experimentally, we have found the reduction of RS-20 and Li-RS-16 after nitrate reduction from XANES/EXAFS, HAADF-TEM EELS. We continue to discuss their stability from the reported Gibbs free energy, standard reduction potential, Pourbaix diagrams, conductivity and valence state change / surface reconstruction in the literature.

Supplementary Table 1 shows that these individual monoxides have different Gibbs free energy as below. From the energy aspect, we can find the stability trend MgO > ZnO > NiO > CoO > CuO. It suggests Cu and Co are more susceptible to surface reduction than MgO. In addition, Calle-Vallejo et al. found the ΔG has a trend CuO > NiO > CoO, based on the reaction $M + H_2O(l) \rightarrow MO + 2(H^+ + e^-)$, indicating that the stability has trend CoO > NiO > CuO[8].

Supplementary Table 2 shows the standard reduction potential from cations to metals. The standard reduction potentials of Cu, Co and Ni cations are more positive than that of Mg and Zn cations. It suggests that Cu, Co and Ni cations are easier to be reduced than Mg and Zn cations.

Moreover, we also used the Pourbaix diagrams to compare the potential stability window[9]. At the condition of pH = 14 and -0.35 V vs. RHE, Mg exists in $Mg(OH)_2$[10]; Co in $HCoO_2^-$[11] or metallic Co[12]; Ni in metallic Ni[13]; Cu in metallic Cu[14]; Zn in $ZnO/Zn(OH)_2$[15]. It also indicates that Co, Ni and Cu are easier to be reduced to metals. Particularly, we also list some recent works on the reduction of Cu/Co compounds during/after nitrate reduction (Supplementary Table 3).

For oxide conductivity, Bérardan et al. reported that $Mg_{0.20}Co_{0.20}Ni_{0.20}Cu_{0.20}Zn_{0.20}O$ has a band gap of ~0.8 eV, and the Li introduction decreases the band gap.[16] It means that Li introduction increases the oxide conductivity. We have attempted to use the two-electrode method by the multimeter and 4-pin probe method by Loresta-GP MCP-T610 (10 mΩ-10 MΩ) to obtain the resistivity of the oxide pellets (Supplementary Fig. 39). However, the resistivities of these three oxides exceed the range of the instrument, which means that these three oxides have poor conductivity.

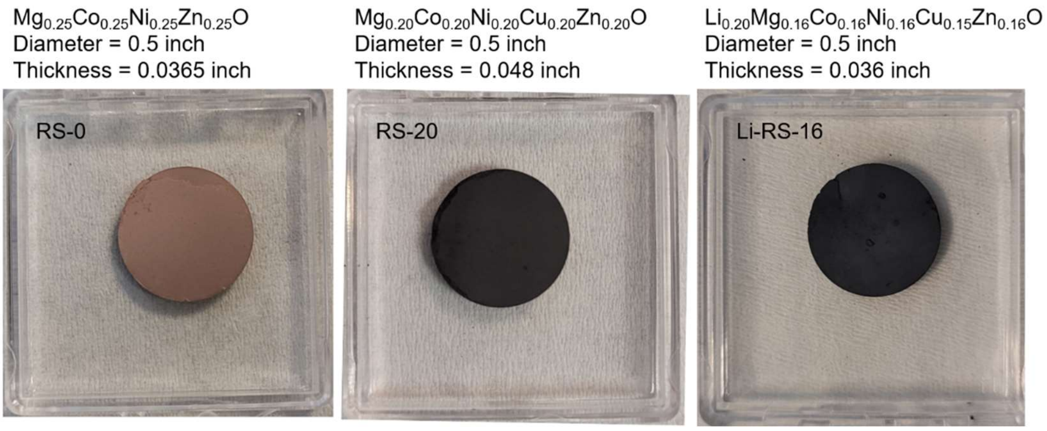

Supplementary Fig. 39 The photo of RS-0, RS-20 and Li-RS-16 pellets for conductivity measurement. To measure the conductivity of RS-0, RS-20 and Li-RS-16, we prepared the oxide pellets without carbon and binder by pressing the raw powders under eight tons followed by heating at 1000 °C for 1 hour with a ramp rate of 5 °C min^{-1}.

Although the electric conductivity is indeed an important factor in reducing the oxide, the situation is different in this work. We also observed the slight metal reduction after nitrate reduction by XANES and EELS. For the electrode used in comparing activity and ammonia yield, carbon was added to mix with these oxides for constructing the conductive network for increase the utilization of those oxides with poor conductivity. The effect from carbon on poorly conductive electrocatalytic materials was previously investigated and reported[17, 18]. When nickel foam is used as the current collector and conductive network, we also observed the metal reduction. Hence, under the existence of the conductive network, we think the conductivity is not the major factor that influence the activity and oxide reduction. Besides, we can also find some important information on the inconsistency between conductivity and bulk oxide reduction from the previous reports and our results. CuO is a p-type semiconductor with a band gap 1.2 eV in bulk and 1.2-2.1 eV in nanostructured CuO.[19] $Mg_{0.20}Co_{0.20}Ni_{0.20}Cu_{0.20}Zn_{0.20}O$, has a band gap ~0.8 V[16]. It means that $Mg_{0.20}Co_{0.20}Ni_{0.20}Cu_{0.20}Zn_{0.20}O$ has a better conductivity than CuO. The cation reduction on CuO during nitrate reduction can be observed obviously[20, 21]. In contrast, the Cu reduction in $Mg_{0.20}Co_{0.20}Ni_{0.20}Cu_{0.20}Zn_{0.20}O$ is slightly. It implies that except for conductivity, under a certain applied potential, other factors such as the oxide composition and crystal structure could affect the oxide reduction.

Supplementary Table 1 ΔH^0 and S^0 and Gibbs free energy

	ΔH^0 / kJ mol^{-1}	S^0 / J mol-1 K^{-1}	ΔG^0 / kJ mol^{-1}
MgO	-601.60	26.95	-609.64
CoO	-237.74	52.85	-253.50
NiO	-246.60	38.58	-258.10
CuO	-156.06	42.59	-168.76
ZnO	-350.46	43.65	-363.47

ΔH^0 and S^0 are the standard enthalpy of formation at 298.15 K and the entropy at 298.15 K, respectively. The Gibbs free energy is calculated according to $\Delta G = \Delta H - T\Delta S$. The values of ΔH^0 and S^0 for thermodynamics were obtained from the database and website[22-24].

Supplementary Table 2 The reduction reaction and standard reduction potential

Reaction	E^0 / V
$Mg^{2+} + 2e^- \rightleftharpoons Mg$	-2.372
$Mg(OH)_2 + 2e^- \rightleftharpoons Mg + 2OH^-$	-2.690
$Co^{2+} + 2e^- \rightleftharpoons Co$	-0.28
$Co(OH)_2 + 2e^- \rightleftharpoons Co + 2OH^-$	-0.73
$Ni^{2+} + 2e^- \rightleftharpoons Ni$	-0.257
$Ni(OH)_2 + 2e^- \rightleftharpoons Ni + 2OH^-$	-0.72
$Cu^{2+} + 2e^- \rightleftharpoons Cu$	0.3419
$Cu(OH)_2 + 2e^- \rightleftharpoons Cu + 2OH^-$	-0.222
$2Cu(OH)_2 + 2e^- \rightleftharpoons Cu_2O + 2OH^- + H_2O$	-0.080
$Cu_2O + H_2O + 2e^- \rightleftharpoons 2Cu + 2OH^-$	-0.360
$Zn^{2+} + 2e^- \rightleftharpoons Zn$	-0.7618
$Zn(OH)_2 + 2e^- \rightleftharpoons Zn + 2OH^-$	-1.249
$ZnO + H_2O + 2e^- \rightleftharpoons Zn + 2OH^-$	-1.260

E^0 represents the standard reduction potential and the value is versus the standard hydrogen electrode. The data in the table refer to the database[22].

Supplementary Table 3 Some cases of Cu/Co reduction during/after the nitrate reduction

Raw catalysts	Under or after reaction	Potential (RHE)	Electrolyte	ref
Cu_2O particles	Cu	<-0.6 V	0.1 M neutral phosphate buffer solution + 0.1 M KNO_3	2023[25]
	Cu and Cu^+	≥ -0.6 V		
Cu_2O cubes	Cu	-0.5 V	0.5 M Na_2SO_4 + 50 mM $NaNO_3$ + NaOH (pH = 10)	2022[26]
CuO nanowire	Cu_2O	-0.45 V	0.5 M Na_2SO_4 solution + 200 ppm nitrate-N	2020[20]
CuO nanobelt	Cu	-0.4 V	1 M KOH + 0.5 M NO_3^-	2021[27]
Cu−Co binary sulfides	Cu/CuO_x-Co/CoO	-0.175 V	0.1M KOH + 0.01M KNO_3	2022[28]
CoO_x	Lower valence Co	−0.3 V	0.1 M KOH +0.1 M KNO_3	2021[29]

Supplementary Table 4 The summary of the selected recent works on electrochemical ammonia generation

	Catalyst	electrolyte	NH_3 FE	NH_3 yield rate	potential	Year / ref
1	$Mg_{0.20}Co_{0.20}Ni_{0.20}Cu_{0.20}Zn_{0.20}O$	1 M KOH + 0.1 M NO_3^-<br>1 M KOH + 0.5 M NO_3^-	99.3%<br>97.2%	5.05 mg mg_{cat}^{-1} h^{-1}<br>26.6 mg mg_{cat}^{-1} h^{-1}	-0.2 V vs. RHE<br>-0.4 V vs. RHE	This work
2	Ru-Cu nanowire	1 M KOH + 0.032 M NO_3	96.0%	76.563 mg h^{-1} cm^{-2}	0.04 V vs. RHE	2022[30]
3	$Cu_{50}Co_{50}$ / nickel foam	1 M KOH + 0.1 M KNO_3	~100%	4.8 mmol cm-2 h^{-1}	-0.2 V vs. RHE	2022[31]
4	$Co_{0.5}Cu_{0.5}$ / carbon fiber	1 M KOH + 0.05 M NO_3^-	>95%	176 mA cm^{-2}	-0.03 V vs. RHE	2022[32]
5	Ar-plasma treated $Cu_{30}Co_{70}$ / carbon paper	1 M KOH + 0.1 M NO_3^-	~80%	5.13 mg cm^{-2} h^{-1}	-0.47 V vs. RHE	2022[33]
6	Cu-Co binary sulfides evolved<br>Cu/CuO_x–Co/CoO hybrids	0.1 M KOH + 0.01 M NO_3^-	93.3%	1.17 mmol cm^{-2} h^{-1}	-0.175 V vs. RHE	2022[28]
7	$ZnCo_2O_4$ / carbon paper	0.1 M KOH + 0.1 M NO_3^-	95.4%	2.10 mg mg^{-1} h^{-1}	-0.4 V vs. RHE	2022[34]
8	CoO/N-doped carbon nanotube/graphite paper	0.1 M KOH + 0.1 M NO_3^-	93.8%	9.04 mg h^{-1} cm^{-2}	-0.6 V vs. RHE	2022[35]
9	Ultrathin CoO_x nanosheets	0.1 M KOH + 0.1 M NO_3^-	93.4%	82.4 mg h^{-1} mg^{-1}	-0.3 V vs. RHE	2021[29]
10	$Cu_{50}Ni_{50}$ / PTFE	1 M KOH + 0.1 M NO_3^-	99%	-	-0.15 V vs. RHE	2020[36]
11	Cu single atom	0.1 M KOH + 0.1 M NO_3^-	84.7%	4.5 mg cm^{-2} h^{-1} / 12.5 mol g_{Cu}^{-1} h^{-1}	-1.0 V vs. RHE	2022[37]
12	Cu(100)-rich rugged Cu-nanobelt	1 M KOH + 0.1 M NO_3	95.3%	650 mmol $h^{-1}$$g_{cat}^{-1}$ h^{-1}	-0.15 V vs. RHE	2021[27]
13	$Cu_{2+1}O$/Ag- carbon cloth	0.1 M KOH + 0.01 M KNO_3	85.03%	2.2 mg h^{-1} cm^{-2}	−0.74 V vs RHE	2023[38]
14	Cu-doped Fe_3O_4	0.1 M KOH + 0.1 M KNO_3	~100%	179.55 mg h^{-1} mg_{cat}^{-1}	−0.6 V vs RHE	2023[39]
15	Cu-modified Ru/C	0.1 M NaOH + 0.1 M $NaNO_3$	95%	23.7 μmol h^{-1} cm^{-2}<br>6.86 mmol h^{-1} mg_{Ru}^{-1}	-0.1 V vs. RHE	2023[40]

16	Cu@nickel foam	1 M KOH + 200 ppm nitrate-N	96.6%	0.252 mmol h^{-1} cm^{-2}	-0.23 V vs. RHE	2021[41]

Supplementary DFT method

Spin-polarized DFT calculations were conducted using plane wave basis sets for valence electrons and projector augmented wave (PAW) potentials for core electrons[42,43], as implemented in the Vienna Ab Initio Simulation Package (VASP)[44,45]. The cutoff energy of plane wave basis sets was set at 450 eV. The Perdew-Burke-Ernzerhof (PBE)[46] functional was employed, where the strong electron correlation effects were evaluated using the Hubbard + U (DFT+U) correction. The U_{eff} parameter was taken from previous DFT studies as follows: $U_{\mathrm{eff}} = 2.0$ eV for Co[47-49] and 3.0 eV for Cu[50,51]. The electronic occupancies were determined through the Gaussian-smearing method with a smearing width of 0.05 eV. Geometry optimization was performed with Monkhorst-Pack k-point meshes of 3 × 3 × 1, using convergence criteria of 1.0×10^{-4} eV for total energy and 0.05 eV/Å for maximum force.

Supplementary References

1. Hibberd, A. M. et al. Co polyoxometalates and a Co_3O_4 thin film investigated by L-edge X-ray absorption spectroscopy. *J. Phys. Chem. C* **119**, 4173-4179 (2015).

2. Kroll, T. et al. X-ray absorption spectroscopy of Na_xCoO_2 layered cobaltates. *Phys. Rev. B* **74**, 115123 (2006).

3. Chin, Y. Y. et al. Spin-orbit coupling and crystal-field distortions for a low-spin $3d^5$ state in $BaCoO_3$. *Phys. Rev. B* **100**, 205139 (2019).

4. Suntivich, J. et al. Estimating hybridization of transition metal and oxygen states in perovskites from O K-edge X-ray absorption spectroscopy. *J. Phys. Chem. C* **118**, 1856-1863 (2014).

5. Wang, Z.L., Yin, J.S. & Jiang, Y.D. EELS analysis of cation valence states and oxygen vacancies in magnetic oxides. *Micron* **31,** 571–580 (2000).

6. Vilá, R. A., Huang, W. & Cui, Y. Nickel impurities in the solid-electrolyte interphase of lithium-metal anodes revealed by cryogenic electron microscopy. *Cell Reports Physical Science* **1,** 100188 (2020).

7. Laffont, L. et al. High resolution EELS of Cu-V oxides: application to batteries materials. *Micron*, **37,** 459-464 (2006).

8. Calle-Vallejo, F., Díaz-Morales, O. A., Kolb, M. J. & Koper, M. T. M. Why is bulk thermochemistry a good descriptor for the electrocatalytic activity of transition metal oxides? *ACS Catal.* **5,** 869-873 (2015).

9. Hochfilzer, D., Chorkendorff, I. & Kibsgaard, J. Catalyst stability considerations for electrochemical energy conversion with non-noble metals: do we measure on what we synthesized? *ACS Energy Lett.* **8,** 1607-1612 (2023).

10. Pesterfield, L. L., Maddox, J. B., Crocker, M. S. & Schweitzer, G. K. Pourbaix (E–pH-M) diagrams in three dimensions. *J. Chem. Educ.* **89,** 891-899 (2012).

11. Garcia, E.M., Santos, J.S., Pereira, E.C. & Freitas, M.B.J.G. Electrodeposition of cobalt from spent Li-ion battery cathodes by the electrochemistry quartz crystal microbalance technique. *J. Power Sources*, **185,** 549-553 (2008).

12. Powell, D., Cortez, J. & Mellon, E. K. A laboratory exercise introducing students to the Pourbaix diagram for cobalt. *J. Chem. Educ.* **64,** 165-167 (1987).

13. Huang, L.-F., Hutchison, M. J., Santucci, R. J., Scully, J. R. & Rondinelli, J. M. Improved electrochemical phase diagrams from theory and experiment: the Ni–water system and its complex compounds. *J. Phys. Chem.* **121,** 9782-9789 (2017).

14. Protopopoff, E. & Marcus, P. Potential–pH diagrams for hydroxyl and hydrogen adsorbed on a copper surface. *Electrochim. Acta* **51,** 408-417 (2005).

15. Borchers, N. et al. Innovative zinc-based batteries. *J. Power Sources* **484,** 229309 (2021).

16. Bérardan, D., Franger, S., Dragoe, D., Meena, A. K. & Dragoe, N. Colossal dielectric constant in high entropy oxides. *Phys. Status Solidi - Rapid Res. Lett.* **10,** 328-333 (2016).

17. Chung, D. Y. et al. Electrokinetic analysis of poorly conductive electrocatalytic materials. *ACS Catal.* **10,** 4990-4996 (2020).

18. Beall, C. E., Fabbri, E. & Schmidt, T. J. Perovskite oxide based electrodes for the oxygen reduction and evolution reactions: the underlying mechanism. *ACS Catal.* **11,** 3094-3114 (2021).

19. Zhang, Q. et al. CuO nanostructures: synthesis, characterization, growth mechanisms, fundamental properties, and applications. *Prog. Mater. Sci.* **60,** 208-337 (2014).

20. Wang, Y., Zhou, W., Jia, R.; Yu, Y. & Zhang, B. Unveiling the activity origin of a copper-based electrocatalyst for selective nitrate reduction to ammonia. *Angew. Chem. Int. Ed.*, **59,** 5350-5354 (2020).

21. Xu, Y. et al. H. Engineering the surface chemical microenvironment over CuO nanowire arrays by polyaniline modification for efficient ammonia electrosynthesis from nitrate. *Appl. Catal. B: Environ.* **320,** 121981 (2023).

22. Haynes, W. M., Lide, D. R. & Bruno, T. J. *Handbook of Chemistry and Physics 97th Edition* (CRC Press, 2016-2017).

23. Archer, D. G., Thermodynamic Properties of Import to Environmental Processes and Remediation. II. Previous Thermodynamic Property Values for Nickel and Some of its Compounds. J. Phys. Chem. Ref. Data **28**, 1485-1507 (1999).

24. NIST Chemistry WebBook. https://webbook.nist.gov/chemistry/

25. Zhou, N. et al. Potential-induced synthesis and structural identification of oxide-derived Cu electrocatalysts for selective nitrate reduction to ammonia. *ACS Catal.* **13,** 7529-7537 (2023).

26. Zhao, J. et al. Constructing Cu-CuO heterostructured skin on Cu cubes to promote electrocatalytic ammonia production from nitrate wastewater. *J. Hazard. Mater.* **439,** 129653 (2022).

27. Hu, Q. et al. Reaction intermediate-mediated electrocatalyst synthesis favors specified facet and defect exposure for efficient nitrate-ammonia conversion. *Energy Environ. Sci.*, **14,** 4989-4997 (2021).

28. He, W. et al. Splicing the active phases of copper/cobalt-based catalysts achieves high-rate tandem electroreduction of nitrate to ammonia. *Nat. Commun.*, **13,** 1129 (2022).

29. Wang, J. et al. Electrocatalytic reduction of nitrate to ammonia on low-cost Ultrathin CoO_x nanosheets. *ACS Catal.*, **11,** 15135-15140 (2021).

30. Chen, F.-Y. et al. Efficient conversion of low-concentration nitrate sources into ammonia on a Ru-dispersed Cu nanowire electrocatalyst. *Nat. Nanotechnol.*, **17,** 759-767 (2022).

31. Fang, J.-Y. et al. Ampere-level current density ammonia electrochemical synthesis using CuCo nanosheets simulating nitrite reductase bifunctional nature. *Nat. Commun.*, **13,** 7899 (2022).

32. Jeon, T. H. et al. Cobalt-copper nanoparticles on three-dimensional substrate for efficient ammonia synthesis via electrocatalytic nitrate reduction. *J. Phys. Chem. C* **126,** 6982-6989 (2022).

33. Wu, A. et al. Boosting electrocatalytic nitrate-to-ammonia conversion via plasma enhanced CuCo alloy–substrate interaction. *ACS Sustain. Chem. Eng.* **10,** 14539-14548 (2022).

34. Huang, P. et al. 3D flower-like zinc cobaltite for electrocatalytic reduction of nitrate to ammonia under ambient conditions. *ChemSusChem*, **15,** e202102049 (2022).

35. Chen, Q. et al. CoO nanoparticle decorated N-doped carbon nanotubes: a high-efficiency catalyst for nitrate reduction to ammonia. *Chem. Commun.*, **58,** 5901-5904 (2022).

36. Wang, Y. et al. Enhanced nitrate-to-ammonia activity on copper-nickel alloys via tuning of intermediate adsorption. *J. Am. Chem. Soc.* **142,** 5702-5708 (2020).

37. Yang, J. et al. Potential-driven restructuring of Cu single atoms to nanoparticles for boosting the electrochemical reduction of nitrate to ammonia. *J. Am. Chem. Soc.* **144,** 12062-12071 (2022).

38. Liu, Y., Yao, X.-M., Liu, X., Liu, Z. & Wang, Y.-Q. $Cu_{2+1}O$/Ag Heterostructure for boosting the electrocatalytic nitrate reduction to ammonia performance. *Inorg. Chem.*, **62,** 7525-7532 (2023).

39. Wang, J. et al. Cu-doped iron oxide for the efficient electrocatalytic nitrate reduction reaction. *Nano Lett.* **23,** 1897-1903 (2023).

40. Chen, J.-Q., Ye, X.-X., Zhou, D. & Chen, Y.-X. Roles of copper in nitrate reduction at copper-modified Ru/C Catalysts. *J. Phys. Chem. C* **127,** 2918-2928 (2023).

41. Li, J. et al. Effect of supporting matrixes on performance of copper catalysts in electrochemical nitrate reduction to ammonia. *J. Power Sources*, **511,** 230463, (2021).

42. Blöchl, P. E. Projector augmented-wave method. *Phys. Rev. B Condens. Matter* **50,** 17953-17979 (1994).

43. Kresse, G. & Joubert, D. From ultrasoft pseudopotentials to the projector augmented-wave method. *Phys. Rev. B* **59,** 1758-1775 (1999).

44. Kresse, G. & Furthmüller, J. Efficiency of ab-initio total energy calculations for metals and semiconductors using a plane-wave basis set. *Comput. Mater. Sci.* **6,** 15-50 (1996).

45. Kresse, G. & Furthmüller, J. Efficient iterative schemes for ab initio total-energy calculations using a plane-wave basis set. *Phys. Rev. B* **54,** 11169-11186 (1996).

46. Perdew, J. P., Burke, K. & Ernzerhof, M. Generalized gradient approximation made simple. *Phys. Rev. Lett.* **77,** 3865-3868 (1996).

47. Sarsari, I. A., Pemmaraju, C. D., Salamati, H. & Sanvito, S. Many-body quasiparticle spectrum of Co-doped ZnO: AGWperspective. *Phys. Rev. B* **87,** 245118 (2013).

48. Kaphle, A., Borunda, M. F. & Hari, P. Influence of cobalt doping on residual stress in ZnO nanorods. *Mater. Sci. Semicond. Process.* **84,** 131-137 (2018).

49. Ciechan, A. & Boguslawski, P. Calculated optical properties of Co in ZnO: internal and ionization transitions. *J. Phys. Condens. Matter* **31,** 255501 (2019).

50. Ma, F. et al. Rational screening of single-atom-doped ZnO catalysts for propane dehydrogenation from microkinetic analysis. *Catal. Sci. Technol.* **10,** 4938-4951 (2020).

51. Ahmad, F., Agusta, M. K. & Dipojono, H. K. Electronic and optical properties of CuO based on DFT+U and GW approximation. *J. Phys. Conf. Ser.* **739,** 012040 (2016).
